# Anisotropic Particle Deposition Kinetics from Quartz Crystal Microbalance Measurements: Beyond the Sphere Paradigm

**DOI:** 10.1021/acs.langmuir.3c03676

**Published:** 2024-04-05

**Authors:** Marta Sadowska, Małgorzata Nattich-Rak, Maria Morga, Zbigniew Adamczyk, Teresa Basinska, Damian Mickiewicz, Mariusz Gadzinowski

**Affiliations:** † Jerzy Haber Institute of Catalysis and Surface Chemistry, Polish Academy of Sciences, Niezapominajek 8, 30-239 Krakow, Poland; ‡ Centre of Molecular and Macromolecular Studies, Polish Academy of Sciences, Henryka Sienkiewicza 112, 90-363 Lodz, Poland

## Abstract

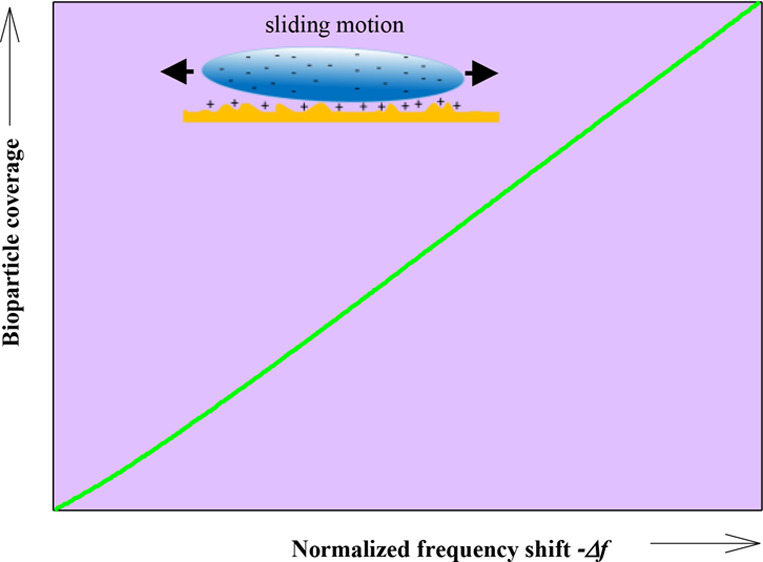

Deposition kinetics of polymer particles characterized by a prolate spheroid shape on gold sensors modified by the adsorption of poly(allylamine) was investigated using a quartz crystal microbalance and atomic force microscopy. Reference measurements were also performed for polymer particles of a spherical shape and the same diameter as the spheroid shorter axis. Primarily, the frequency and dissipation shifts for various overtones were measured as a function of time. These kinetic data were transformed into the dependence of the complex impedance, scaled up by the inertia impedance, upon the particle size to the hydrodynamic boundary layer ratio. The results obtained for low particle coverage were interpolated, which enabled the derivation of Sauerbrey-like equations, yielding the real particle coverage using the experimental frequency or dissipation (bandwidth) shifts. Experiments carried out for a long deposition time confirmed that, for spheroids, the imaginary and real impedance components were equal to each other for all overtones and for a large range of particle coverage. This result was explained in terms of a hydrodynamic, lubrication-like contact of particles with the sensor, enabling their sliding motion. In contrast, the experimental data obtained for spheres, where the impedance ratio was a complicated function of overtones and particle coverage, showed that the contact was rather stiff, preventing their motion over the sensor. It was concluded that results obtained in this work can be exploited as useful reference systems for a quantitative interpretation of bioparticle, especially bacteria, deposition kinetics on macroion-modified surfaces.

## Introduction

Experimental studies of particle deposition kinetics furnish essential information about their interactions with interfaces, especially the adhesion strength, which is a crucial issue for colloid science, biophysics, medicine, soil chemistry, etc. This knowledge can be used to control and optimize various practical processes, such as filtration, flotation, protective coating formation, paper making, catalysis, and bioengineering. In addition, results acquired for colloid systems under well-defined transport conditions can serve as useful reference results for the interpretation of macromolecule and bioparticle adsorption phenomena, especially virus and bacteria attachment to abiotic surfaces.

It should be underlined that the shape of colloid silica particles,^1−4^ carbon nanotubes,^5,6^ polymer microparticles,^7−9^ or macroion molecules^10−13^ resembles cylinders or prolate spheroids. Similarly, the anisotropic molecular shape is common among biocolloids, such as DNA fragments,^14−16^ proteins,^17,18^ viruses,^19−22^ and bacteria,^23−26^ comprising the common bacterial strains, such as *Escherichia coli*, *Legionella pneumophila*, *Sphingopyxis alaskensis*, or *Hylemonella gracilis*.^27,28^

Because of its significance, nano- and microparticle adsorption kinetics was extensively studied by numerous experimental techniques, such as atomic force microscopy (AFM),^29−31^ scanning electron microscopy (SEM),^32−35^ ellipsometry,^36−38^ reflectometry,^39−41^ surface plasmon resonance (SPR),^42,43^ and electrokinetic methods.^44,45^

In comparison to these techniques, the widely used quartz crystal microbalance (QCM) method exhibits pronounced advantages, enabling sensitive, *in situ* measurements of deposition/desorption kinetics under flow conditions for particle size ranging from nanometers^46−53^ to a few micrometers.^54,55^ However, these investigations were mainly focused on spherical particles, with only a few studies performed for anisotropic particles. In refs (56−59), the deposition of liposomes was investigated and theoretically interpreted in terms of lattice Boltzmann numerical modeling. It was predicted that the absolute value of the negative frequency shift decreased with the coverage and axis ratio of the liposomes modeled as oblate spheroids with the size of 78 nm.

Scarce experiments were reported for colloid particles of an elongated shape. In ref (60), the deposition of rod-shaped polystyrene particles with the aspect ratio of 2:1 and 4:1 at silica sensors modified by adsorption of poly-l-lysine (PLL) was studied by a QCM. The frequency shift acquired for the third overtone was used to determine the initial deposition rate of the particles as a function of the ionic strength (varied between 0.05 and 50 mM). It was confirmed that the deposition rate was considerably lower for the spheroidal particles compared to the spherical particles, independent of the ionic strength.

Similar measurements were performed in ref (61), where the deposition kinetics of native and stretched 200 nm in diameter polystyrene particles at alginate and harpeth humic-acid-coated silica sensors was investigated. A significant decrease in the absolute value of the frequency shifts was observed for the more rough sensor coated by harpeth humic acid, especially for the stretched particles characterized by the aspect ratio of 1.6. However, these interesting experimental results were not interpreted by any theoretical model.

In a recent work,^62^ the adsorption kinetics of positively charged polymer particles of a spheroidal shape at bare gold and silica sensors was investigated using the QCM method. It was confirmed that, for such particles, the hydrodynamic slip effect played a significant role, decreasing manifold the QCM signals compared to firmly attached particles with respect to both the frequency and dissipation shifts.

A few investigations were also performed for bioparticles of anisotropic shape, such as protein molecules^63−71^ and bacteria.^23−28^ In refs (69 and 70), adsorption kinetics of fibrinogen, whose molecule has an elongated shape with the length to width aspect ratio exceeding 7, was studied by QCM. The experimental results were interpreted in terms of a coarse-grained Monte-Carlo-type theoretical modeling, which allowed the determination of the ratio of the real coverage to that calculated from the Sauerbrey equation.

Interesting studies based on the QCM were also reported for bacteria strains, *inter aliaE. coli*, *Salmonella*,^25−27^ and soil bacteria.^71,72^ Whereas in most of the works a negative frequency shifts were measured, in ref (26), dealing with *Lactococcus lactis*, a positive, albeit rather small, equal to 20 Hz frequency shift (third overtone, polystyrene-coated sensor) was reported. However, no attempt was undertaken to quantify the obtained results and to elucidate physical mechanisms of bacteria attachment.

One can argue that the scientific value of these tedious experiments could be enhanced if appropriate reference results obtained for well-characterized particles and sensor surfaces were available. Therefore, considering the lack of such experimental data, the main goal of this work was to determine the mechanism of prolate spheroidal particle deposition at solid/electrolyte interfaces with the main focus on assessing the feasibility of the QCM measurements to furnish quantitative kinetic results. Experiments were performed for polymer particles bearing a negative charge analogous to most bioparticles, especially bacteria.^27,28^ To facilitate a reliable interpretation of the results, the gold sensors were thoroughly characterized in terms of topographical properties, comprising the average surface height, the root mean square (rms), and the skewness. Analogous deposition kinetic measurements were carried out for spherical particles of the size matching the shorter spheroid axis. The real, often referred to as the dry, particle coverage was determined by atomic force microscopy (AFM) and in a continuous manner, applying a hybrid random sequential adsorption (RSA) modeling.

This enabled us to quantitatively determine the complex impedance of the sensor as a function of the ratio of the particle size to the hydrodynamic boundary layer thickness for a broad range of coverage. In the case of spheroidal particles, the real and complex impedance components were equal to each other and linearly decreased with the square root of the overtone number. This was interpreted as the effect of a hydrodynamic, lubrication-like contact with the sensor, enabling a sliding motion of the particles. In contrast, the spherical particle contact was rather stiff, preventing their motion over the sensor.

One can suppose that the obtained results can be used as useful reference data for estimating the range of applicability of recent hydrodynamic theories of QCM sensor impedance in the case of a heterogeneous, particle-like load.^73−76^

## Experimental Section

### Materials

All chemical reagents comprising sodium chloride, sodium hydroxide, and hydrochloric acid were commercial products of Sigma-Aldrich and were used without additional purification. Ultrapure water was obtained using the Milli-Q Elix and Simplicity 185 purification system from Millipore.

### Synthesis of Poly(styrene/α-*tert*-butoxy-ω-vinylbenzyl-polyglycidol) [P(S/PGL)] Spheroidal Microparticles

Synthesis of P(S/PGL) spheroidal microparticles was described in ref (77). It was a tedious process consisting of three main steps: (i) synthesis of the α-*tert*-butoxy-ω-vinylbenzyl-polyglycidol (PGL) macromonomer, (ii) synthesis of P(S/PGL) microspheres using styrene and the PGL macromonomer, and (iii) preparation of spheroidal P(S/PGL) particles from the spherical particles applying the stretching of poly(vinyl alcohol) (PVA) films containing embedded P(S/PGL) microspheres.

The particle chemical composition was characterized by X-ray photoelectron spectroscopy (XPS), performed using the PHl 5000 VersaProbe, scanning electron spectroscopy for chemical analysis (ESCA) microprobe (ULVAC-PHI, Japan/U.S.A.) instrument at a base pressure below 5 × 10^–9^ mbar. The particle morphology and size distribution were characterized by scanning electron microscopy (SEM) using a JEOL 5500LV apparatus (Akishima, Japan).

Except for the spheroidal P(S/PGL), negatively charged polystyrene particles supplied by Invitrogen were used as a control solute in the deposition kinetics measurements carried out by a QCM.

Freshly cleaved mica sheets were used in the AFM measurements of spheroid adsorption kinetics under diffusion conditions.

The poly(allylamine) (PAH) aqueous solutions prepared from the crystalline powder supplied by the PSS Polymer Standards Service were used for the modification of the mica and QCM sensors.

Gold substrate plates used in the streaming potential measurements were prepared using the Si/SiO_2_ wafers as the support coated with the gold layer (approximately 100 nm thick) by thermal evaporation (see the Supporting Information).

Gold sensors with a 5 MHz fundamental frequency used in the experiments were supplied by Quartz Pro, Järfälla, Sweden. Before measurements, the sensors were cleaned in a mixture of 96% sulfuric acid (H_2_SO_4_) and hydrogen peroxide (30%) in a volume ratio of 1:1 for 10 min. Afterward, the sensors was rinsed by ultrapure water and boiled at 80 °C for 30 min, rinsed again with ultrapure water, and dried out in a stream of nitrogen gas.

The topography of the sensors was determined by AFM imaging carried out under ambient conditions in semi-contact mode. All of the relevant parameters were determined (see the Supporting Information). The rms of the gold sensors was equal to 1.4 ± 0.1 nm, and the roughness correlation length and wavelength were 50 ± 5 and 70 ± 5 nm, respectively.

### Methods

The bulk concentration of particles in the stock suspension was determined by the dry mass method. Before each deposition experiment, the stock suspension was diluted to the desired concentration, typically equal to 40–200 mg L^–1^ by pure NaCl solutions with the pH adjusted to 5.6.

The diffusion coefficient of the particles was determined by dynamic light scattering (DLS) using the Zetasizer Nano ZS instrument from Malvern. The hydrodynamic diameter was calculated using the Stokes–Einstein relationship. The electrophoretic mobility of particles was measured by the laser Doppler velocimetry (LDV) technique using the same apparatus. The zeta potential was calculated using the Ohshima formula^78^ (see the Supporting Information).

The QCM measurements were carried out according to the standard procedure described in ref (52) using the Q-Sence QCM Instrument (Biolin Scientific, Stockholm, Sweden). First, a stable baseline of a pure electrolyte with a fixed concentration (either 1 or 10 mM NaCl and pH 5.8) was attained in the QCM-D cell for a defined flow rate (typically 1 × 10^–3^ cm^3^ s^–1^). Next, a PAH solution of the bulk concentration equal to 5 mg L^–1^ was flown through the cell until a stable signal were obtained. Afterward, the particle suspension of a fixed concentration was flushed at the same flow rate. After a prescribed time, the desorption run was initiated, where a pure electrolyte solution of the same pH and ionic strength was flushed through the cell.

The deposition kinetics of particles was determined using the AFM method as previously described in ref (52). Accordingly, after completion of a QCM adsorption run, the sensor was removed from the suspension and imaged under ambient conditions by AFM using the NT-MDT Solver BIO device with the SMENA SFC050L scanning head. The number of particles per unit area (typically 1 μm^2^) denoted hereafter by *N*, was determined by a direct counting of over a few equal-sized areas randomly chosen over the sensor with the total number of particles exceeding 2000.

The direct AFM enumeration method was also used to quantify the adsorption kinetics of the spheroidal particles on mica. The aim of these experiments was to determine, independent of the dry weight method, the real particle concentration in the suspension after the dilution step and to determine the maximum particle coverage used as a scaling variable in the RSA modeling.

The zeta potential of bare and PAH-covered plates was determined via streaming potential measurements performed according to the procedure described in ref (8) applying the Smoluchowski formula, where the correction for the surface conductivity was considered (see the Supporting Information).

All experiments have been performed at the temperature of 298 K.

The deposition kinetics of particles was theoretically interpreted in terms of a hybrid approach, where the bulk particle transport was described by the convective–diffusion equation with the nonlinear boundary condition derived from the random sequential model (a detailed description of this approach is given in the Supporting Information).

## Results and Discussion

### Bulk Particle and Substrate Characteristics

The relevant physicochemical characteristics of the spheroidal SA200 and spherical L200 particles used in the QCM kinetic measurements are collected in Table 1. The former particles were characterized by the dimension 2*a* × 2*b* × 2*b* of 1010 × 205 × 205 nm (derived from AFM) and 1020 × 215 × 215 (derived from SEM, where a ca. 10 nm thick layer was sputtered), with a low dispersity of ca. 5%. Hence, their axis ratio *As* = *a*/*b* and the cross-section area in the side-on orientation *S*_g_ were equal to 4.93 and 0.163 μm^2^. The diffusion coefficient of the particles directly measured by DLS was equal to 1.1 × 10^–12^ m^2^ s^–1^ (for the NaCl concentration of 1–10 mM). This yields the hydrodynamic diameter of 430 nm calculated from the Stokes–Einstein formula. The zeta potential of the particles derived from the LDV measurements varied between −49 and −36 mV for the NaCl concentration range of 1–10 mM and pH 5–6.

**Table 1:**
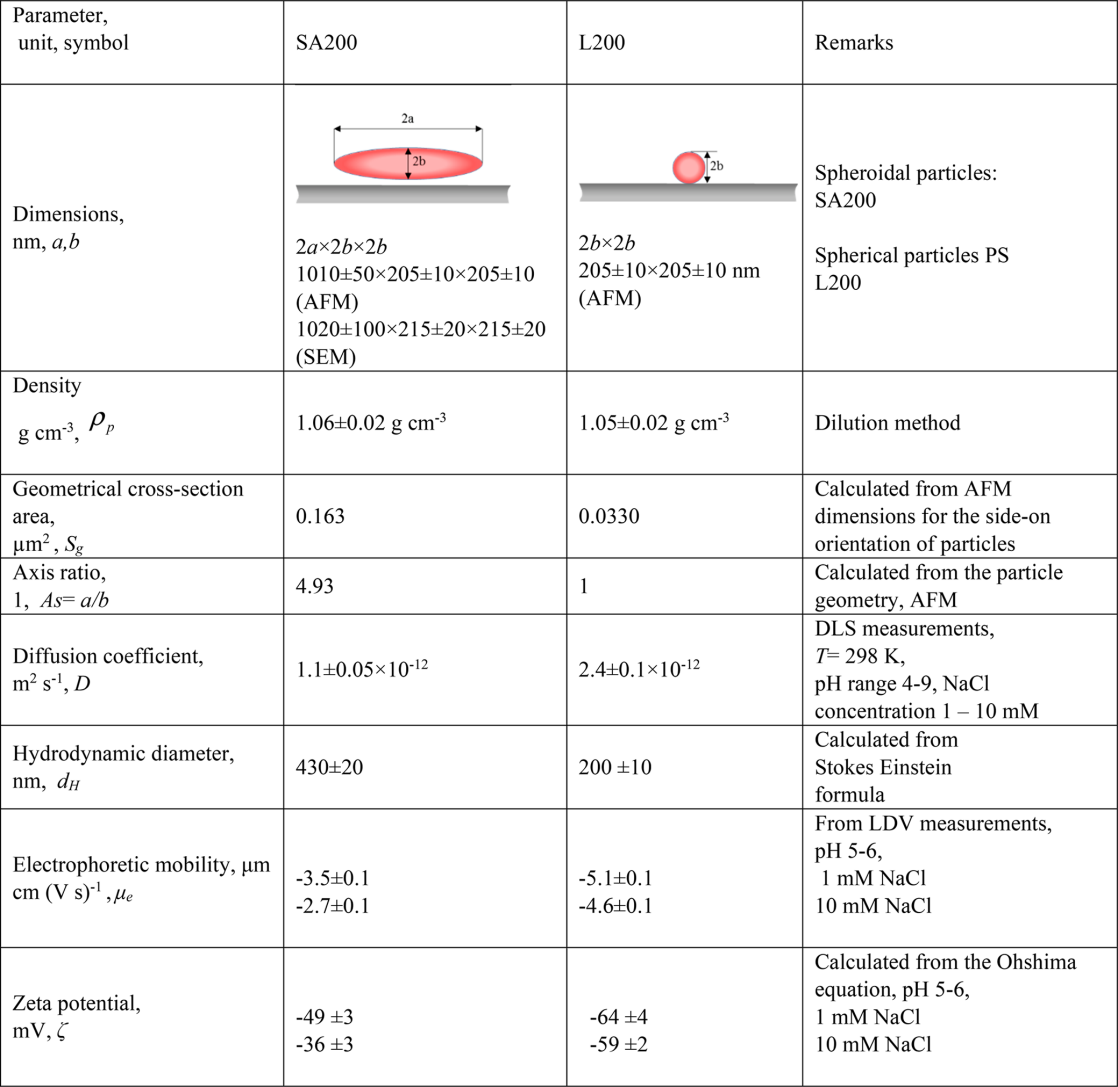
Physicochemical Characteristics of the Spheroidal Particles (SA200) and Polystyrene Spherical Particles (L200) Investigated in This Work

In an analogous way, the L200 particles were characterized by AFM, DLS, and LDV measurements. Their diffusion coefficient was equal to 2.4 × 10^–12^ cm^2^ s^–1^ (for the pH range of 5–6 and the above NaCl concentration range). This corresponds to the hydrodynamic diameter of 200 nm, which coincides within error bounds with the shorter axis of the spheroidal SA200 particles. The zeta potential of the particles was equal to −64 and −59 for the NaCl concentration range of 1–10 mM and pH 5–6.

The zeta potential of gold substrates was equal to −58 and −47 mV at 1 and 10 mM NaCl concentration, respectively, at pH equal to 5–6, whereas the zeta potential of PAH-covered gold and silica substrates was in both cases equal to 60 and 40 mV for this NaCl concentration range and pH 5–6.

### Small Particle Coverage Limit

A series of QCM kinetic runs yielding the frequency and dissipation shifts were performed for the SA200 and L200 particles under the same physicochemical conditions, i.e., 1 mM NaCl, pH 5.6, volumetric flow rate of 10^–3^ cm^3^ s^–1^, and various bulk suspension concentrations. After completion of the run, the real particle coverage was determined by AFM, as described above. This parameter was used as a scaling variable for the hybrid RSA modeling, which furnished the particle mass coverage versus the adsorption time dependencies in a continuous manner (see the Supporting Information). Such a procedure, previously applied in ref (52), enabled a reliable determination of the impedance and other derivative functions used in the literature for the interpretation of the QCM measurements.^65,68,73,74^

**Figure 1:**
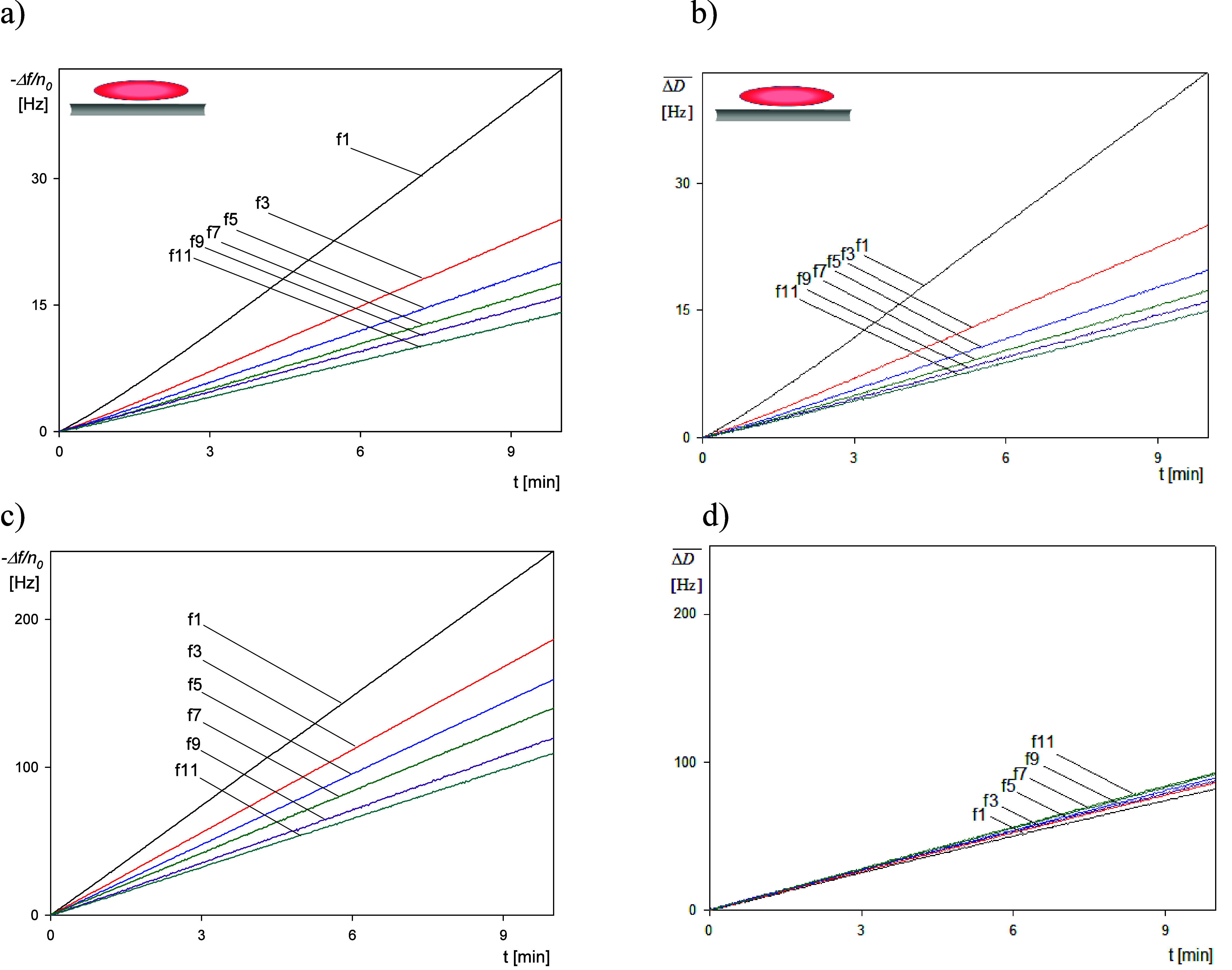
Short-time deposition kinetic results derived from QCM: (a) frequency shift −Δ*f*/*n*_o_ versus deposition time for the overtones 1–11, SA200, spheroidal particles, (b) bandwidth shift  versus deposition time for the overtones 1–11, SA200 spheroidal particles, (c) frequency shift −Δ*f*/*n*_o_ for the overtones 1–11, L200, spherical particles, and (d) bandwidth shift  for the overtones 1–11, L200, spherical particles. Experimental conditions: PAH-modified gold sensor, 1 mM NaCl, pH 5.6, volumetric flow rate of 10^–3^ cm^3^ s^–1^, and bulk particle concentration of 200 mg L^–1^.

Primary results derived from the QCM kinetic measurements are shown in Figure 1 for the short time period up to 10 min. In panels a and b, the normalized frequency shifts −Δ*f*/*n*_o_ and the bandwidth shifts  (where *f*_F_ is the fundamental frequency of the sensor of 5 × 10^6^ Hz and Δ*D* is the dissipation shift) are plotted as a function of time for the spheroidal particles and for various overtones *n*_o_ (1–11). Analogously, in panels c and d, the dependencies of the frequency and bandwidth shifts for the spherical particles are shown. Although quantitatively different, in all cases, the frequency and bandwidth shifts can be approximated by linear functions of the deposition time. This facilitates a precise calculation of the complex impedance ΔZ* in the limit of low particle coverage considering that it is defined as the ratio of the tangential stress (i.e., the force per the surface area of the sensor) to the surface velocity^55,75^
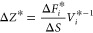
1where Δ*F*_i_^*^ is the excess complex force transferred to the sensor because of the particles present in its vicinity (but not necessarily adsorbed), Δ*S* is the surface area of the sensor, and *V*_i_^*^ is the sensor complex velocity.

It should be mentioned that, in a general case, the impedance depends upon the particle size, shape, orientation, sensor topography (roughness), oscillation frequency, etc., which renders its *ab initio* theoretical calculation a complicated task, even for spherical particles.^73−76^ However, the impedance can be experimentally determined using the above QCM kinetic data, taking advantage of the following definitions^53,59,74^
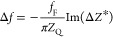
2
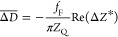
3where *Z*_Q_ is the acoustic impedance of quartz equal to 8.8 × 10^6^ kg m^–2^ s^–1^.

For a purely inertia load, the impedance is given by
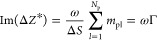
4
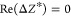
where ω = 2π*f*_F_*n*_o_ is the angular velocity of the sensor oscillations, *n*_o_ is the overtone number, *m*_pl_ is the single-particle mass, *N*_p_ is their number at the surface, and
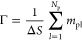
5is the net mass of the particle layer per unit area referred to as the mass surface coverage or just coverage.

It should be mentioned that eq 4 is valid for an arbitrary particle shape, size, and coverage. Using this inertia load impedance, i.e., ωΓ, as a scaling variable,^55,73^ one obtains the following expressions connecting the normalized impedance with the frequency and bandwidth shifts:
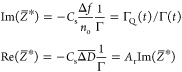
6where
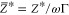

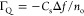
7is the QCM coverage referred to often as the “wet” mass
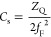
8is the Sauerbrey constant equal to 0.177 mg m^–2^ Hz^–1^ for *f*_F_ = 5 × 10^6^ Hz, and
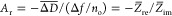
9is the acoustic ratio.^73,74^

Assuming that Γ_Q_ and Γ are a linear function of the time, which was the case for the runs shown in Figure 1, eq 6 can be converted to the following form:
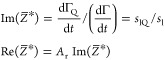
10where *s*_lQ_ and *s*_l_ are the corresponding slopes of Γ_Q_ and Γ on the deposition time dependencies, respectively.

**Figure 2:**
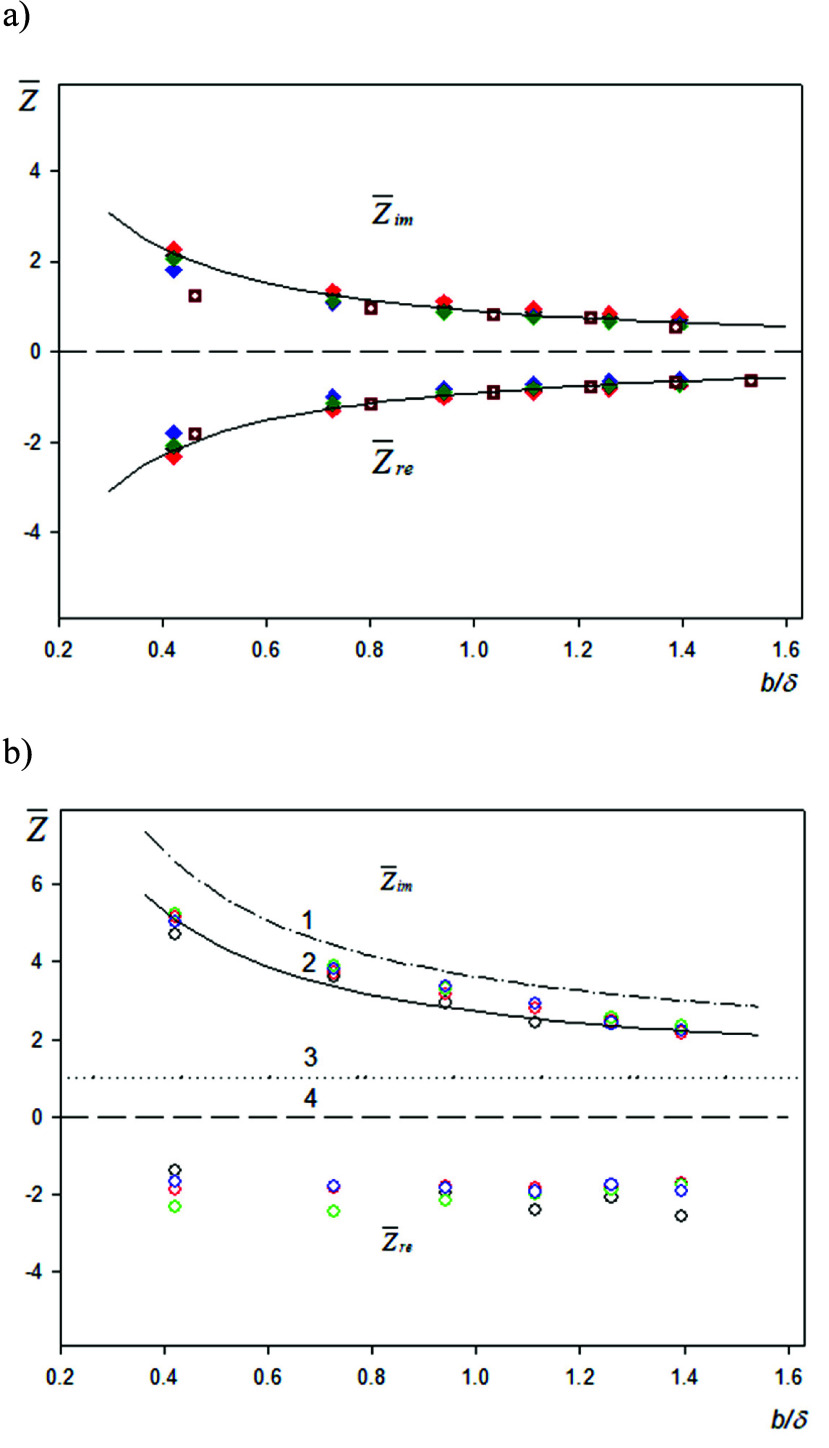
Imaginary and real parts of the sensor impedance in the limit of low particle coverage determined from the experimental kinetic data. (a) Spheroidal particles: the points 
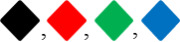
 show experimental results derived from measurements carried out for anionic spheroids under various bulk concentrations and deposition times, and the square points show the previous results obtained in ref (62) for cationic spheroids on a bare gold sensor. Experimental conditions: PAH-modified gold sensor, 1 mM NaCl, pH 5.6, and volumetric flow rate of 10^–3^ cm^3^ s^–1^. The solid line shows the theoretical values of the impedances calculated from eq 12. (b) Spherical particles (L200): the points 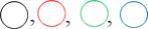 show experimental results derived from measurements carried out under various bulk concentrations and deposition times. Experimental conditions: PAH-modified gold sensor, 1 mM NaCl, pH 5.6, and volumetric flow rate of 10^–3^ cm^3^ s^–1^. The dashed/dotted line 1 shows the theoretical values calculated from eq 13 using the Tarnapolsky and Freger^55^ model pertinent to large *b*/δ; the solid line 2 shows the results calculated from the fitting function eq 14; the dotted line 3 shows the results pertinent to the purely inertia load; and the dashed line 4 shows the zero impedance.

In Figure 2, the dependencies of the imaginary and real impedance components on the *b*/δ parameter are shown for spheroidal (panel a) and spherical particles (panel b), where
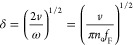
11is the hydrodynamic boundary layer thickness and *v* is the dynamic viscosity of the fluid.

Considering that, for the anionic spheroids, *b* = 103 nm, the *b*/δ parameter in experiments whose results are shown in Figure 2a varied between 0.433 and 1.44. One should also mention that, because of the roughness of the gold sensor (a thorough analysis of the sensor topography is presented in the Supporting Information), the energy minimum distance *h*_m_ of the spheroids was equal to 5 nm. This gives 0.05 for the *h*_m_/*b* parameter pertinent to these experiments.

The average values of *Z̅*_im_ calculated from the experimental results shown in Figure 2a were equal to 2.1 ± 0.1 and 0.67 ± 0.05 for *b*/δ of 0.433 and 1.44, respectively. The average values of *Z̅*_re_ were equal to −2.1 ± 0.1 and −0.70 ± 0.05 for *b*/δ of 0.433 and 1.44, respectively, which practically coincide (in absolute terms) with *Z̅*_im_. This regularity was also observed for the intermediate values of the *b*/δ parameter, and for the cationic (positively charged) spheroid deposition on the bare gold sensor previously reported in ref (62), see Figure 2a. This indicates that, for spheroidal particles, the complex impedance is proportional to 1 – *i*. Considering this, the experimental results were fitted by the following function:
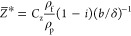
12with the dimensionless constant *C*_z_ equal to 0.96.

One should underline, however, given the soft hydrodynamic contact of the particles with the sensor, that this constant is expected to depend upon the *h*_m_/*b* parameter.

As seen in Figure 2a, eq 12 well reflects the experimental data for the entire range of the *b*/δ parameter.

Analogous results obtained for the spherical particles, where the range of *b*/δ was practically the same as for spheroids, are shown in Figure 2b. One can notice that the *Z̅*_im_ impedance component was considerably larger than that for spheroids and was equal to 5.0 ± 0.1 and 2.1 ± 0.05, for *b*/δ of 0.433 and 1.44, respectively. Therefore, in comparison to spheroids, it did not decrease below unity, which was marked as the dotted line in Figure 2b. On the other hand, the real impedance component was practically independent of *b*/δ within experimental error bounds and assumed an average value of −2.0 ± 0.2. Such a behavior was predicted in ref (74) for a stiff contact of the particles with the sensor, although theoretical data pertinent to combined hydrodynamic and inertia forces are not available. However, some theoretical predictions for a stiff contact can be derived from the model of a freely oscillating sphere formulated by Tarnapolsky and Freger,^55^ yielding the following expression for *Z̅*_im_ valid for *b*/δ larger than unity:
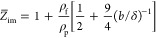
13The theoretical predictions derived from eq 13, shown in Figure 2b as the dashed line, overestimate the experimental data, although the general trend is well-reflected for the entire range of the *b*/δ parameter. A better fit can be achieved using the following formula:
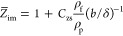
14where *C*_zs_ = 1.8.

It is also interesting to mention that the acoustic ratio −*Z̅*_re_/*Z̅*_im_ calculated using eq 9 considering that *Z̅*_re_ = −2 agrees within experimental error bounds with the acoustic ratio defined in ref (74) (considering the scaling factor of ^1^/_2_).

Moreover, using the above fitting functions, one can formulate the following expression enabling direct calculations of the real spheroidal particle coverage using the experimental frequency and bandwidth shifts:
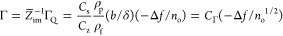
15where
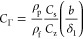
16and δ_1_ is the boundary layer thickness for the first overtone. In our case, *C*_z_ was equal to 0.96 for *h*_m_/*b* = 0.05. It should be noticed that, according to eq 15, the real particle coverage is proportional to −Δ*f*/*n*_o_^1/2^.

Analogously, using the experimental bandwidth, one obtains the formula
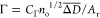
17For spherical particles under the stiff contact regime, one has
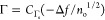
18where
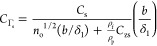
19With the advantage of the fact that eqs 16 and 18 are valid under the linear kinetic regime, they can be directly used to determine the mass transfer constant, a parameter of basic significance for every kinetic study.^44,79,80^ Considering the definition of the mass transfer constant, one can derive the following expression for the spheroids and spheres, respectively:
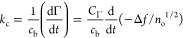
20
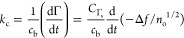
21where *c*_b_ is the bulk mass concentration of the particles, expressed in mg L^–1^.

**Figure 3:**
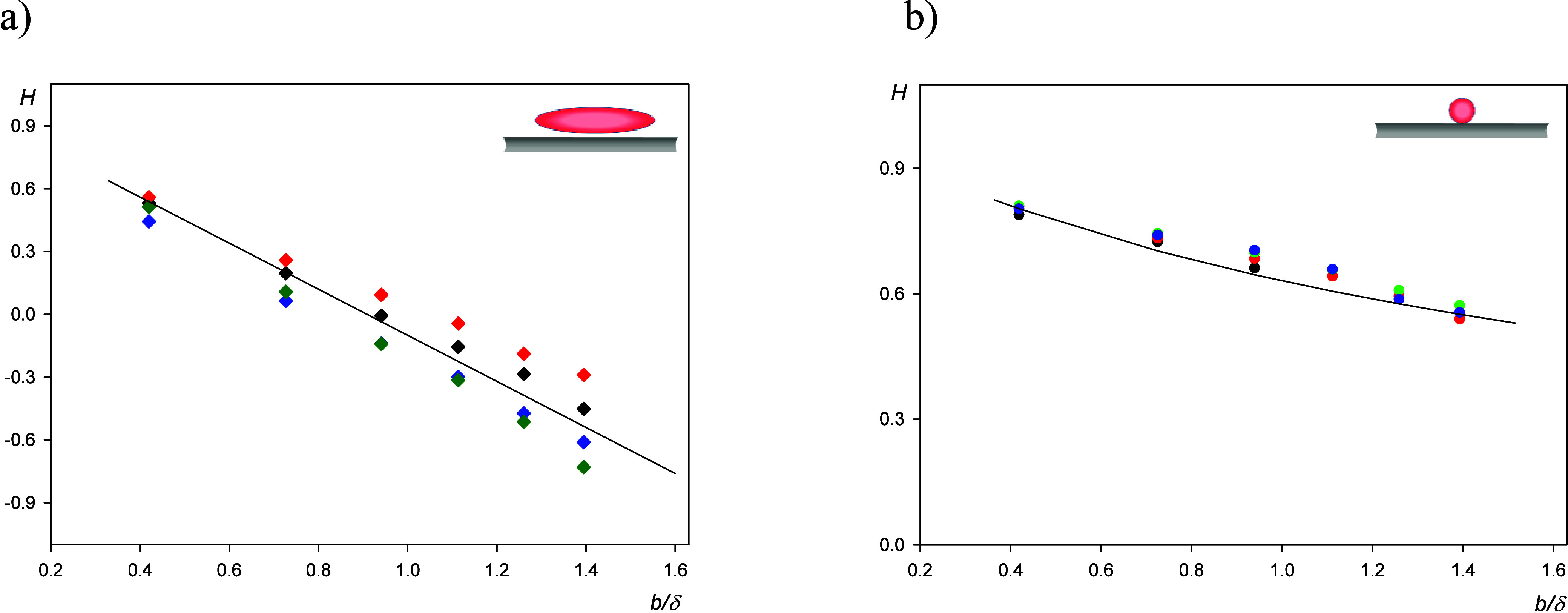
Dependence of the *H* function in the limit of low particle coverage upon the *b*/δ parameter. (a) Spheroidal particles: the solid line denotes the theoretical data calculated using eq 24. (b) Spherical particles: the solid line denotes the theoretical data calculated using eq 25. Experimental conditions are the same as in panels a and b of Figure 2.

Additionally, knowing the *Z̅*_im_ impedance component, one can calculate the correction function *H* often used in the literature^52,65,66,69,70,74^ for the interpretation of experimental results derived from QCM measurements and defined as
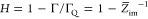
22Thus, knowing *H*, one can calculate the real particle coverage as
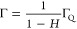
23The dependencies of *H* on the *b*/δ parameter for spheroids and spheres are shown in panels a and b of Figure 3, respectively. In the former case, the function was equal to 0.48 at *b*/δ = 0.433 and then monotonically decreased, attaining negative values for *b*/δ larger than 0.8. This trend is well-reflected by the following fitting function derived using eq 12 for spheroids:
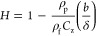
24In the case of spheres, the function was equal to 0.80 at *b*/δ = 0.433 and then slowly decreased, attaining 0.54 at *b*/δ = 1.44. Using eq 14, the following formula was derived to interpolate the experimental data:
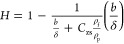
25As mentioned, all of these formulas are strictly valid for the low coverage regime, where the frequency and bandwidth shifts remain a linear function of the deposition time. In the next section, these results are extended to the arbitrary particle coverage, exploiting the long-time kinetic data derived from the QCM measurements.

### Analysis of the Long-Time Kinetic Data

**Figure 4:**
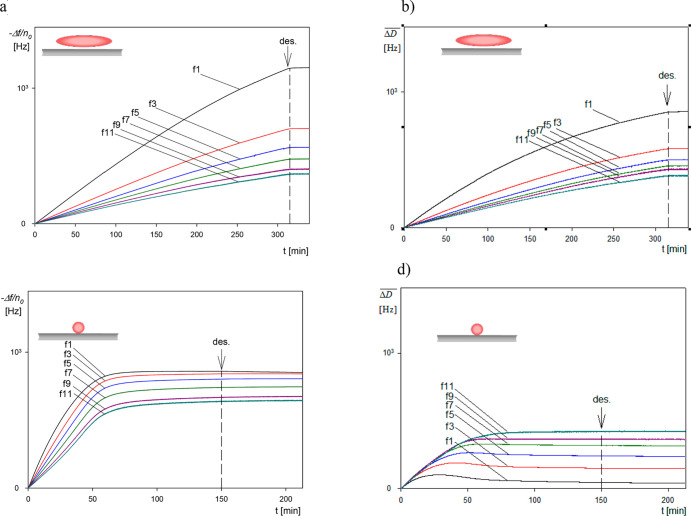
Long-time deposition kinetic results derived from QCM: (a) frequency shift −Δ*f*/*n*_o_ versus deposition time for the overtones 1–11, SA200, spheroidal particles, (b) bandwidth shift  versus deposition time for the overtones 1–11, SA200 spheroidal particles, (c) frequency shift −Δ*f*/*n*_o_ for the overtones 1–11, L200, spherical particles, and (d) bandwidth shift  for the overtones 1–11, L200, spherical particles. Experimental conditions: PAH-modified gold sensor, 1 mM NaCl, pH 5.6, volumetric flow rate of 10^–3^ cm^3^ s^–1^, and bulk particle concentration of 200 mg L^–1^. The arrows show the initiation of the desorption run.

The long-time kinetic runs performed under identical conditions as before (see legend to Figure 1) are shown in Figure 4. In panels a and b, the normalized frequency shifts −Δ*f*/*n*_o_ and bandwidth shifts  are shown for spheroidal particles and various overtones. Analogously, in panels c and d, the dependencies of the frequency and bandwidth shifts for spherical particles are shown. It is worth mentioning that no change in the signals was observed after the initiation of the desorption run, where a pure electrolyte of the same ionic strength was flushed through the QCM cell.

**Figure 5:**
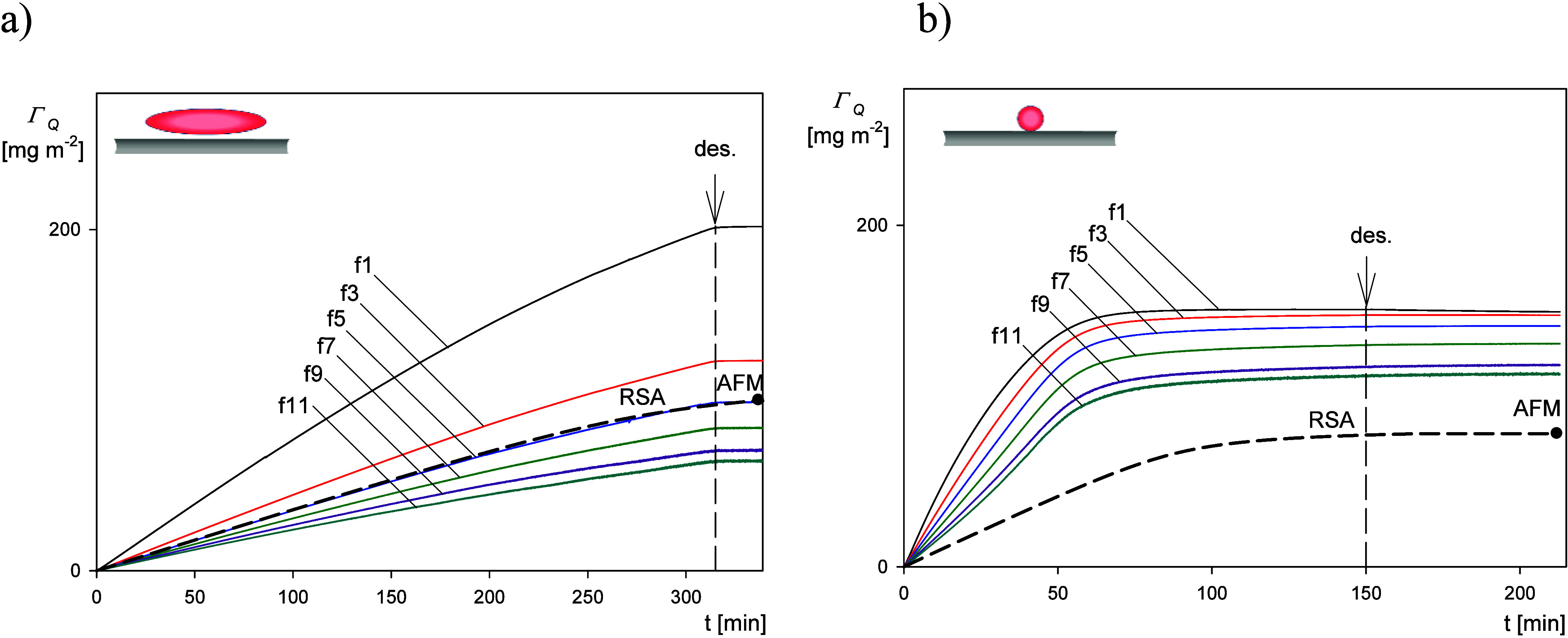
Kinetics of particle deposition expressed as the dependence of the QCM coverage calculated using the Sauerbrey constant for the overtones 1–11. The points represent the real (dry) particle coverage derived from AFM, and the dashed lines denote the theoretical results derived from the hybrid RSA model. (a) Spheroidal particles. (b) Spherical particles. Experimental conditions are the same as in Figure 4. The arrows show the initiation of the desorption run.

These primary experimental data were converted to the QCM coverage calculated as −*C*_s_(Δ*f*/*n*_o_), yielding the kinetic runs shown in Figure 5 for spheroids and spheres. In both cases, the QCM coverage, which significantly exceeded the real coverage for the first overtone, systematically decreased for larger overtones. Interestingly, in the case of spheroids, the kinetic curve derived from QCM coincided for the fifth overtone with that pertinent to the real coverage derived from the RSA calculations. It should be mentioned that the RSA kinetics was calibrated using the AFM coverage determined after finishing each measurement, comprising the desorption step, using a direct counting procedure. The maximum AFM coverage was equal to 100 and 78 mg m^–2^ for spheroids and spheres, respectively.

**Figure 6:**
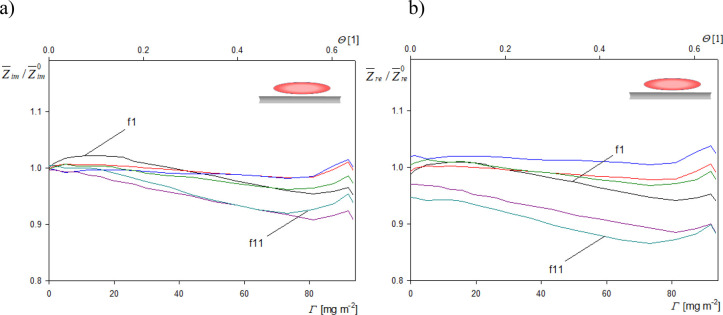
Dependence of the (a) imaginary and (b) real impedance components (normalized using the initial impedances) on the real particle coverage for spheroidal particles. The upper horizontal axis shows the dimensionless particle coverage . Experimental conditions are the same as in Figure 4.

Using the experimental results shown in Figure 5, the imaginary and real impedance components can be calculated from eq 6 as a function of the deposition time (with the real particle coverage as a scaling parameter). To facilitate the analysis of obtained results, the kinetic dependencies obtained in this way are converted to the dependencies of *Z̅*_im_/*Z̅*_im_^0^ and *Z̅*_re_/*Z̅*_re_^0^ on the real particle coverage (where *Z̅*_im_^0^ and *Z̅*_re_^0^ are the above determined impedances in the limit of low particle coverage). The results shown in Figure 6 for spheroids confirm that the normalized imaginary and real impedances were close to unity for all overtones and for the entire range of particle coverage. This is a significant result showing that the previously derived formula describing the impedance components, i.e., eq 12, remains valid for an arbitrary coverage of spheroids. As a consequence, eqs 15 and 16 can be used to calculate the deposition kinetics, expressed in terms of the real coverage, using the experimental frequency or bandwidth shifts acquired for various overtones.

The above results obtained for spheroids are consistent with a non-localized deposition mechanism, where the particles may undergo sliding motions in the direction parallel to the sensor surface without physically contacting the sensor surface.^8,52^ A thorough analysis of the interactions of a spheroid with the rough substrate (Supporting Information) showed that this is physically feasible because of the presence of a deep energy minimum appearing at the distance of 5 nm. This prevented the diffusion of the captured particles in the perpendicular direction over the distance larger than a fraction of a nanometer. On the other hand, the particle motion in the parallel direction was feasible because the spheroidal particle length significantly exceeded the average distance between the roughness peaks, estimated to be about 200 nm. In effect, the particles were deposited at a few peaks (see Figure S6a of the Supporting Information) and could freely execute a sliding motion along the surface. Thus, their interactions with the surface were purely hydrodynamic of lubrication-like type, controlled by the smallest distance between the particle and the sensor surfaces, which appeared close to the center of the particle. Because these hydrodynamic interactions were confined to a small space underneath the particles, the dependence of the impedance upon the coverage is expected to be rather negligible.

It should be mentioned that an analogous behavior was recently reported in ref (81). It was shown that spherical particles (of the size between 25 and 100 nm) linked to the sensor by a molecular receptor showed a perfectly linear increase in the normalized frequency shift with the dimensionless coverage θ up to 0.5. This is tantamount to the fact that the normalized sensor impedance was independent of the particle coverage, which was observed in Figure 6 for spheroids.

On the other hand, for spherical particles, whose size was comparable to the average distance between roughness peaks, their perpendicular and parallel motions were prohibited because this would require a significant increase in the energy (see the Supporting Information). Therefore, their contact with the surface was rather stiff, enabling the transfer of the inertia force appearing because of the sensor surface periodic acceleration.

**Figure 7:**
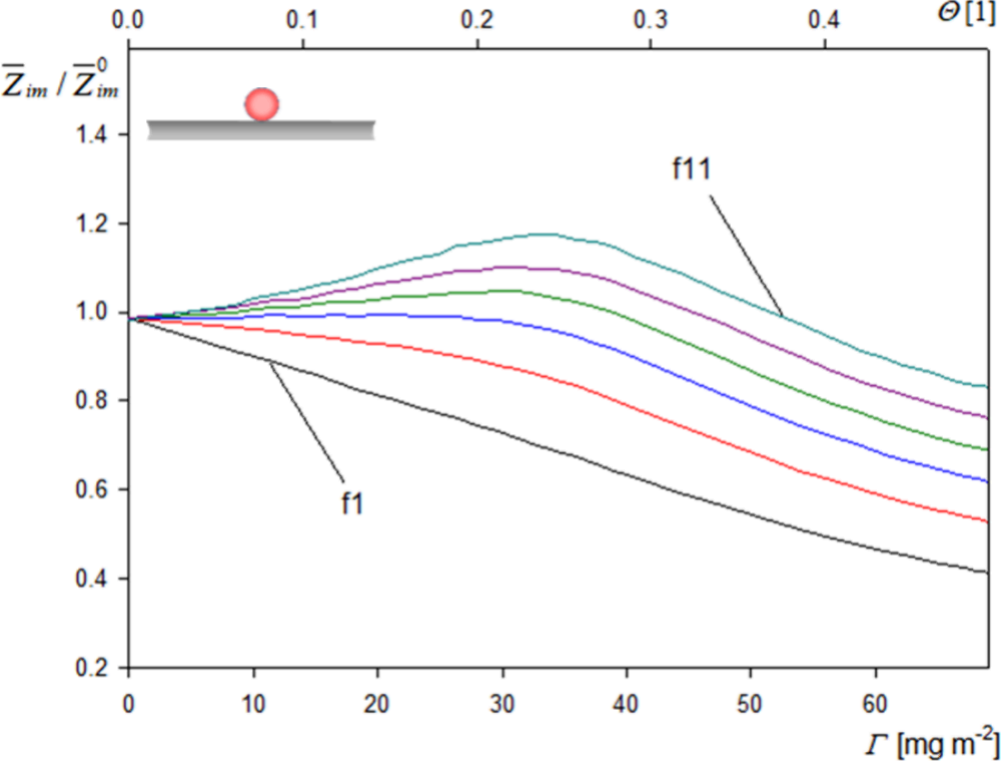
Dependence of the imaginary impedance components upon the real particle coverage for spherical particles. The upper horizontal axis shows the dimensionless particle coverage . Experimental conditions are the same as in Figure 4.

**Figure 8:**
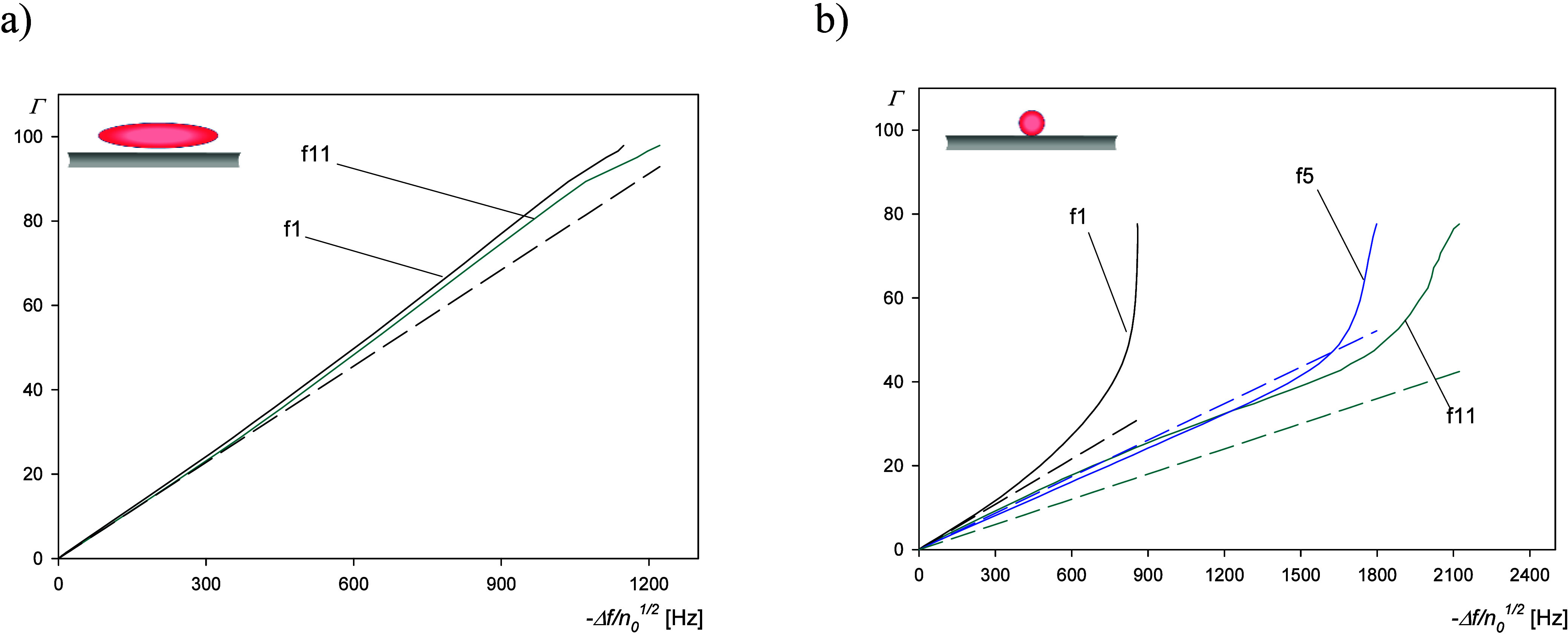
Real spheroidal particle coverage versus −Δ*f*/*n*_o_^1/2^ for different overtones. (a) Spheroidal particles: the dashed line shows the results calculated from eqs 15 and 16. (b) Spherical particles: the dashed line shows the results calculated from eqs 18 and 19. Experimental conditions are the same as in Figure 4.

Because of such stiff contact of spherical particles, the dependence of the normalized imaginary impedance component upon the real particle coverage, shown in Figure 7, was more complicated in comparison to this dependence found for spheroids. For the first to fifth overtones, it monotonically decreased with the real particle coverage, but at higher overtones, these dependencies were non-monotonic, exhibiting a maximum at the coverage of ca. 42 mg m^–2^ (θ = 0.30). Unfortunately, in this case, no universal formula can be derived, furnishing the real particle coverage for arbitrary frequency shifts. The range of applicability of eq 15 for spheroids and eq 18 for spheres can be estimated analyzing the results shown in panels a and b of Figure 8 as the dependencies of the real particle coverage on −Δ*f*/*n*_o_^1/2^. As expected for spheroids, eq 15 yields adequate results for the wide range of −Δ*f*/*n*_o_^1/2^ and for all overtones. On the other hand, for spheres, eq 18 only yields an adequate fit for the fifth overtone and −Δ*f*/*n*_o_^1/2^ smaller than 1500 Hz. For other overtones, the range of applicability of eq 18 is rather limited. One should also consider that the choice of an adequate overtone, yielding the best results, cannot be done *a priori* without performing a thorough analysis of experimental data. Therefore, the results obtained for spheres suggests that, at the present time, the deconvolution of the QCM signal aimed at the calculation of the real coverage is only feasible in the limit of low-frequency shifts.

## Conclusion

Deposition kinetics of anionic spheroidal particles at polycation-modified gold sensors was determined applying a QCM supplemented by AFM and analyzed in terms of theoretical modeling based on the hybrid RSA approach. Analogous measurements were carried out for spherical particles of the same diameter as the spheroid shorter axis.

A thorough analysis of the kinetic data enabled determination of the complex impedance for various overtones, which corresponded to the *b*/δ parameter between 0.433 and 1.44 and for a broad range of the real particle coverage.

It was shown that the frequency shifts for all overtones were linear functions of the particle coverage. These results were explained in terms of a hydrodynamic, lubrication-like contact of particles with the sensor, enabling their sliding motion.

The feasibility of such a non-localized deposition mechanism of spheroids was confirmed by the analysis of their interactions with the sensor surface whose topography was thoroughly characterized by AFM. Considering the experimental results, a Sauerbrey-like equation, eq 15, was derived enabling a facile determination of the real spheroidal particle coverage using the frequency or bandwidth (dissipation) shifts derived from experiments. This equation can also be used to determine the mass transfer constant of the particles in the QCM cell.

On the other hand, for spherical particles, the imaginary impedance significantly exceeded unity for all overtones and arbitrary coverage range and the frequency shifts were nonlinear functions of the particle coverage, which was interpreted as the effect of a stiff contact. On the basis of these results, eq 18 was derived enabling calculation of the real particle coverage and as a consequence of the mass transfer rate constant. However, it was shown that this equation only yields precise data for low-frequency shifts.

One can expect that the results obtained in this work can be used as useful reference systems for a quantitative interpretation of bioparticle, especially bacteria, deposition phenomena, significantly extending the applicability range of the QCM technique.

## Supporting Information Available

Synthesis and characteristics of spheroidal particles, modification of gold substrate by PAH adsorption, topographical characteristics of sensors, modeling deposition kinetics: the hybrid RSA approach, and calculation of the Derjaguin–Landau–Verwey–Overbeek (DLVO) energy (PDF)
