# Supplementary material for: Anisotropic Particle Deposition Kinetics from Quartz Crystal Microbalance Measurements: Beyond the Sphere Paradigm
Source: Langmuir. 2024 Apr 5;40(15):7907–19. doi: 10.1021/acs.langmuir.3c03676 (PMC11025136; doi:10.1021/acs.langmuir.3c03676)
Supplement: Supplementary file 1 — la3c03676_si_001.pdf [file la3c03676_si_001.pdf]

SUPPORTING INFORMATION

Anisotropic Particle Deposition Kinetics from QCM: Beyond the Sphere Paradigm

Marta Sadowska[1*], Małgorzata Nattich-Rak[1], Maria Morga[1], Zbigniew Adamczyk[1]*, Teresa Basinska[2], Damian Mickiewicz[2], Mariusz Gadzinowski[2]

1. Jerzy Haber Institute of Catalysis and Surface Chemistry, Polish Academy of Sciences, Niezapominajek 8, 30 - 239 Krakow, Poland;
2. Centre of Molecular and Macromolecular Studies, Polish Academy of Sciences, Henryka Sienkiewicza 112, 90-363 Lodz, Poland;

*Corresponding authors, e-mails: zbigniew.adamczyk@ikifp.edu.pl,
marta.sadowska @ikifp.edu.pl

Table of contents:

1. Synthesis and Characteristics of Spheroidal Particles
2. Modification of Gold Substrate by PAH Adsorption
3. Topographical Characteristics of Sensors
4. Modeling Deposition Kinetics-the Hybrid RSA Approach
5. Calculation of DLVO Energy

1. Synthesis and Characteristics of Spheroidal Particles

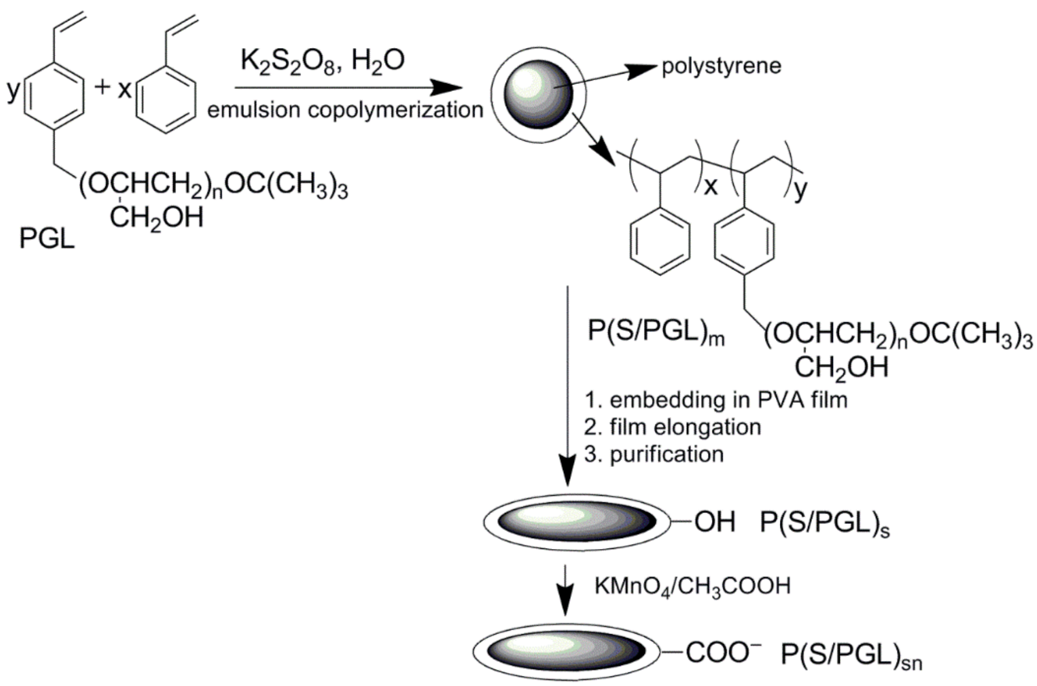

Figure S1. Synthesis scheme of the P(S/PGL) spheroidal particles. Explanations of abbreviations: PGL – polyglycidol macromonomer, P(S/PGL)m – poly(styrene/polyglycidol) microspheres, P(S/PGL)s – poly(styrene/polyglycidol) spheroids, P(S/PGL)sn – poly(styrene/polyglycidol) spheroids negatively charged.

Preparation of spheroidal particles (PS/PGL) consisted of three stages: (i) synthesis of α-*tert*-butoxy-ω-vinylbenzyl-polyglycidol (PGL) macromonomer, (ii) synthesis of P(S/PGL) microspheres using styrene and PGL macromonomer, (iii) preparation of spheroidal particles P(S/PGL) from the spherical ones. Poly(styrene/α-*tert*-butoxy-ω-vinylbenzyl-polyglycidol) (P(S/PGL)) spheroidal microparticles were produced by stretching poly(vinyl alcohol) (PVA) films containing embedded P(S/PGL) microspheres [1,2]. The synthesis scheme is schematically shown in Scheme S1.

1.1. Synthesis of α-*tert*-butoxy-ω-vinylbenzyl-polyglycidol (PGL) macromonomer

The process consisted of three steps: synthesis of 1,1-ethylethoxyglycidyl ether in reaction of glycidol and ethyl vinyl ether [3], anionic polymerization of 1,1-ethylethoxyglycidyl ether initiated with potassium *tert*-butoxide and terminated with p-chloromethylstyrene, hydrolysis of ethyl ethoxy groups in poly(1,1-ethylethoxyglycidyl ether) was performed using $AlCl_3 \times 6H_2O$, according procedure described by Namboodiri [4] and subsequently applied by Halacheva et al. [5]. The process yielded poly(α-*tert*-butoxy-ω-

vinylbenzyl-polyglycidol) macromonomer. The macromonomer structure, molecular weight and dispersity were confirmed by ^{1}H NMR spectra of macromonomer before and after hydrolysis of ethyl ethoxy groups and GPC traces, respectively.

In ^{1}H NMR spectrum of poly[α-*tert*-butoxy-ω-vinylbenzyl-poly(1-ethylethoxy-glycidyl ether)] the following signals were observed: 7.38 (m) protons in phenyl ring; 6.71 (m) proton in vinyl group; 5.25 (d) and 5.80 (d) $–CH_2$; 4.60 (d) $–C_6H_5–\underline{CH_2}–O$; 3.40-3.75 (m) $–OCH$, $–OCH_2$; 1.17 (m) $–C(CH_3)_3$, $–CHCH_3$, $–OCH_2CH_3$, whereas, after hydrolysis of ethyl ethoxy groups in ^{1}H NMR spectrum signals with the following chemical shifts (in ppm) assigned to protons in PGL macromolecule have been registered: 7.38 (m) protons in phenyl ring; 6.71 (m) proton in vinyl bond; 5.78 (d) and 5.23 (d) $=CH_2$; 4.60 (d) $–C_6H_5–CH_2–O$; 3.45-3.64 (m)$–CH<$, $–CH_2O–$; 1.12 (s) $−C(CH_3)_3$. The spectrum did not contain signals of ethyl ethoxy groups which confirmed the full removal of blocking components. Integration of $-C(CH_3)_3$ (at 1.12 (s)) and of $–CH<$ and $–CH_2O−$ (overlapping signals in the range 3.45-3.64) allowed calculation of M_n of PGL macromonomer. M_n of PGL macromonomer was equal to 2560 g mol^{-1}. The M_n value was confirmed by end-group ($(CH_3)_3C–$) and main-chain groups ($–CH_2CH(CH_2OH)O–$) analysis of the polymer in ^{1}H NMR spectrum which is shown together with assignment of signals in Figure S2.

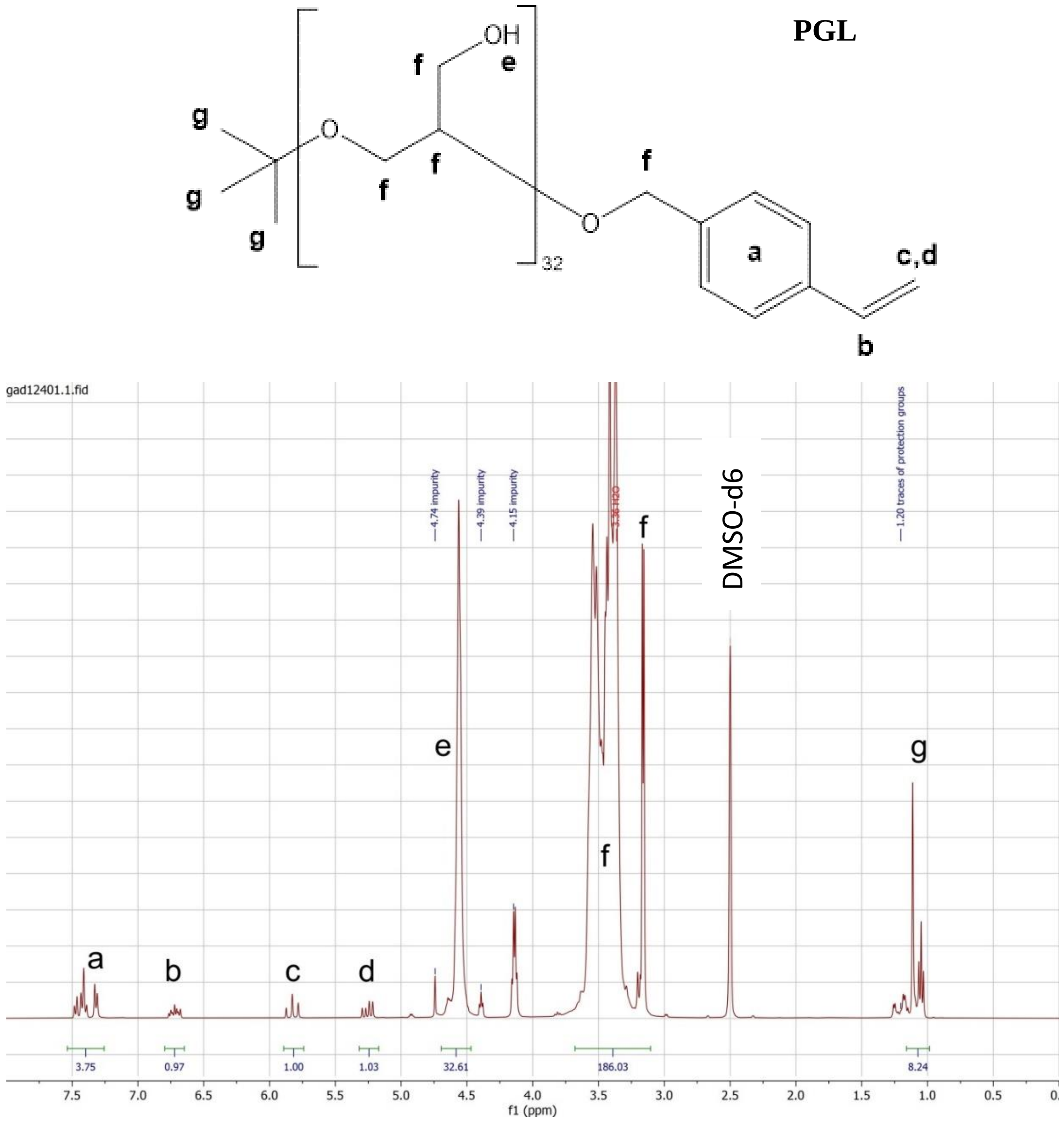

Figure S2. ^{1}H NMR spectrum of PGL macromonomer used for preparation of P(S/PGL) particles. Spectrum for PGL registered in DMSO-d6 using Bruker NMR spectroscopy (400 MHz).

1.2. Preparation of poly (styrene / α - *tert* - butoxy - ω - vinylbenzyl - polyglycidol) microspheres ($P(S/PGL)_m$)

Poly(styrene/α-*tert*-butoxy-ω-vinylbenzyl-polyglycidol) microspheres ($P(S/PGL)_m$) were synthesized by emulsion polymerization of styrene and α-*tert*-butoxy-ω-vinylbenzyl-polyglycidol macromonomer (PGL). The details of synthesis of $P(S/PGL)_m$ were described in Refs.[6,7]. Briefly, 0.25 g of PGL macromonomer ($M_n = 2560$ g mol^{-1}) dissolved in 120 ml of distilled water and then styrene (10 g) were placed in glass reactor, degassed by purging with argon and stirred at room temperature. After 60 minutes of stirring, temperature was raised to 70°C and 0.216 g of $K_2S_2O_8$ was added. The polymerization was carried out under argon, at 70°C for 27 h with stirring at 380 rpm. Then, the microspheres were purified by

centrifugation, washed with 10^{-3} M HCl and then, with new portions of water. The washing steps were repeated four times. Diameters of microspheres were determined by analysis of scanning electron microscopy (SEM) microphotographs and DLS. Number average diameters of microspheres (D_n) and diameter dispersity index (denoted as D_w/D_n) were calculated on a basis of measurements of diameters of 500 microspheres (randomly chosen from different SEM microphotographs).

1.3. Preparation of poly(styrene/α-*tert*-butoxy-ω-vinylbenzylpolyglycidol) spheroidal particles $P(S/PGL)_{sn}$

The microspheres P(S/PGL) (11.0 g of 5.7% suspension) were mixed with (100 g) aqueous solution of PVA (12.5% w/w). The 22.9 g of the mixture was transferred to PTFE mold with the size 7x9 cm and stored at 22°C for 4 days to evaporate water. The dried films were removed from the mold and cut into stripes with the size 1x8 cm. Then, the thickness of the stripes every 1 cm along their length was measured using thickness tester with accuracy ±1 μm (Sylvac μs 229). In the next step, the PVA stripes with embedded particles were uniaxially stretched in a chamber with controlled temperature, using Instron apparatus (ITW). After equilibration at 120°C, the two stripes were fixed in two clamps, fasten at the top and at the bottom of the chamber. The size of stripes, which underwent elongation were 1×6 cm plus additional 1 cm at each side for catching by clamps. Then, the stripes were elongated with a constant speed of 10 mm/min. till the required length of the stripe (equal 15 cm) was obtained. When the temperature in a heating chamber decreased to ca. 50 °C, the stretched stripes of the PVA film were removed from the clamps and cooled to the room temp. Next, the length and thickness of the film stripes were measured again. In the final step, the particles were recovered by dissolving the PVA matrices in di. water followed by repeated centrifugation in a centrifuge (MPW380) and multiple exchange of supernatant for di. water. Every 2-3 washings the supernatant was checked for the presence of PVA by measuring its surface tension.

The elongation process yields uncharged spheroidal particles because of the presence of PVA within the particles surface layer. In order to prepare negatively charged particles surface hydroxyl groups were oxidized to carboxyl groups. The surface modification procedure was as follows: a suspension of $P(S/PGL)_s$ (20 ml, 18 mg/ml) was mixed with 4.0 ml of acetic acid (3.0 M) and 6.0 ml of $KMnO_4$ solution (0.05 M) for 45 min, at 20ºC. Then, 6.0 ml of ascorbic acid (0.05 M) was added to reduce precipitated MnO_2 and the

particles were centrifuged several times in order to replace supernatant for di. water. Representative image of negatively charged P(S/PGL)spheroids is presented in Figure S3.

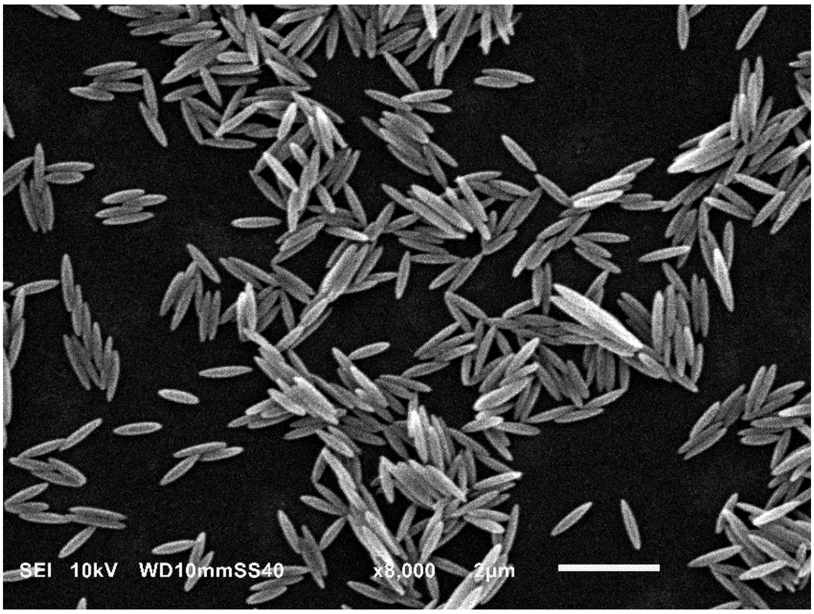

Figure S3. The SEM image of the $(PS/PGL)_{sn}$ anionic spheroidal particles. Average particle dimensions $2a \times 2b \times 2b$: 1020±100×215±20×215±20 nm, where *a, b* are the longer and shorter spheroid axes, respectively.

The diffusion coefficient of the particles was directly measured as a function of pH for different ionic strengths using the dynamic light scattering (DLS). Then, using the diffusion coefficient data, the hydrodynamic diameter of the particles was calculated using the Stokes-Einstein formula. It was practically independent of pH and ionic strength assuming an average value of 430±20 nm.

On the other hand, the electrophoretic mobility of particles for different ionic strengths and pHs was directly measured by the LDV (Laser Doppler Velocimetry) using the Zetasizer Nano (Malvern Instruments, Great Britain). The zeta potential ζ was calculated from the following formula derived in Ref. [8] pertinent to cylindrical particles where the double-layer polarization effect was considered:

$$\mu_e = \frac{\varepsilon\zeta}{3\eta} + \frac{\varepsilon\zeta}{3\eta} f_1(\kappa c) - \frac{4\varepsilon\zeta}{9\eta}\left(\frac{e\zeta}{kT}\right)^2 [f_3(\kappa c) + f_4(\kappa c)] \tag{S1}$$

where:

$$f_1(\kappa c) = 1 + \left(1 + \frac{2.55}{\left[\kappa c\left(1+e^{-\kappa c}\right)\right]}\right)^{-2}$$

$$f_3(\kappa c) = \frac{\kappa c\left(\kappa c + 0.162\right)}{2\left[\left(\kappa c\right)^3 + 9.94\left(\kappa c\right)^2 + 18.7\kappa c + 0.147\mathrm{e}^{-9.41\kappa c}\right]} \qquad \text{(S2)}$$

$$f_4(\kappa c) = \frac{1}{2}\left(m_+ + m_-\right)\frac{9\kappa c\left(\kappa c + 0.361\mathrm{e}^{-0.475\kappa c} + 0.0878\right)}{8\left[\left(\kappa c\right)^3 + 10.8\left(\kappa c\right)^2 + 18.2\kappa c + 0.0633\right]}$$

ε is the electric permittivity and η is the dynamic viscosity of the electrolyte, $\kappa^{-1} = \left(\frac{\varepsilon kT}{2e^2 I}\right)^{1/2}$ is the Debye screening length (double-layer thickness), k is the Boltzmann constant, T is the absolute temperature, $e = 1.602\times10^{-19}$ C is the elementary charge, I is the ionic strength of the electrolyte solution, m_+, m_- are the dimensionless ion drag coefficients and c is the cylinder radius.

To calculate the zeta potential, the implicit dependence represented by Eq.(S1) was numerically inverted for a set of experimentally measured electrophoretic mobility values. The obtained data are given in Table 1 of the main manuscript.

2. Modification of Gold Substrate by PAH Adsorption

The zeta potentials of the bare gold and silica/gold layers functionalized by the PAH adsorption (mimicking the QCM sensor structure) were acquired by the streaming potential measurements carried out according the previously applied procedure [9,10] in a parallel-plate microfluidic channel. Several runs were performed at four different pressure differences that allowed us to obtain the slope of the streaming potential vs. hydrostatic pressure dependence. Using the slope the zeta potential of the surface was calculated from the Smoluchowski equation. The pH of the PAH solution was 5.6, the bulk concentration 5 mg L^{-1} and the flow volumetric flow rate 0.35 cm^3 s^{-1}. To prevent the PAH macroion depletion, all glassware was preconditioned three times with the macroion solutions of the same concentration as that used in the experiments.

The dependence of the zeta potential of silica/gold substrate on the adsorption time of PAH is shown in Figure S4. As can be seen, the negative zeta potential of the bare silica/gold

substrate rapidly increased and attained after 20 min plateau values of 60 and 43 mV for the NaCl concentration of 1 and 10 mM, respectively.

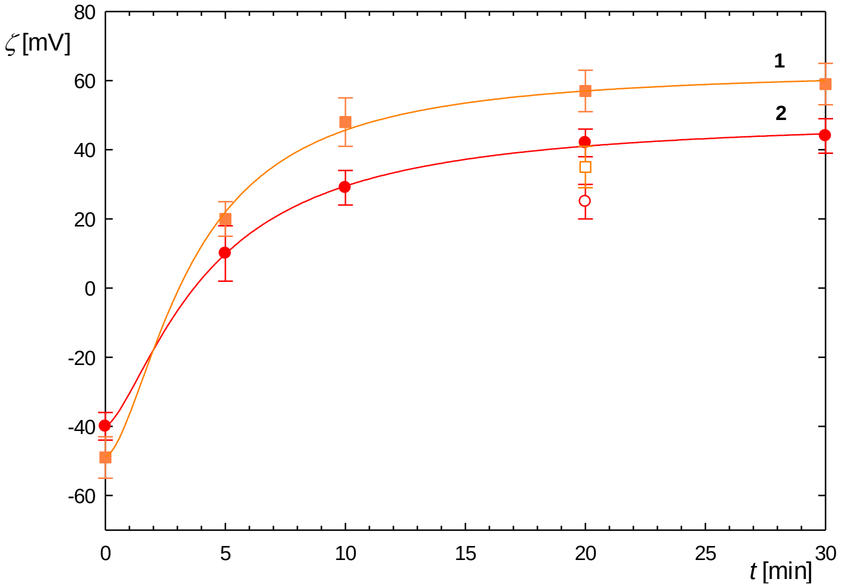

Figure S4. The zeta potential of the silica/gold layer vs the time of PAH adsorption in the streaming potential cell, pH 5.6, bulk PAH concentration 5 mg L^{-1}, solution flow rate 0.35 cm^3 s^{-1}, curve 1: 1 mM NaCl, curve 2: 10 mM NaCl.

3. Topographical Characteristics of Sensors

The topography of the sensors used in QCM measurements was determined by atomic force microscopy (AFM) imaging carried out under ambient conditions in a semi-contact mode. A typical gold sensor image and the corresponding height profiles are shown in Figure S5.

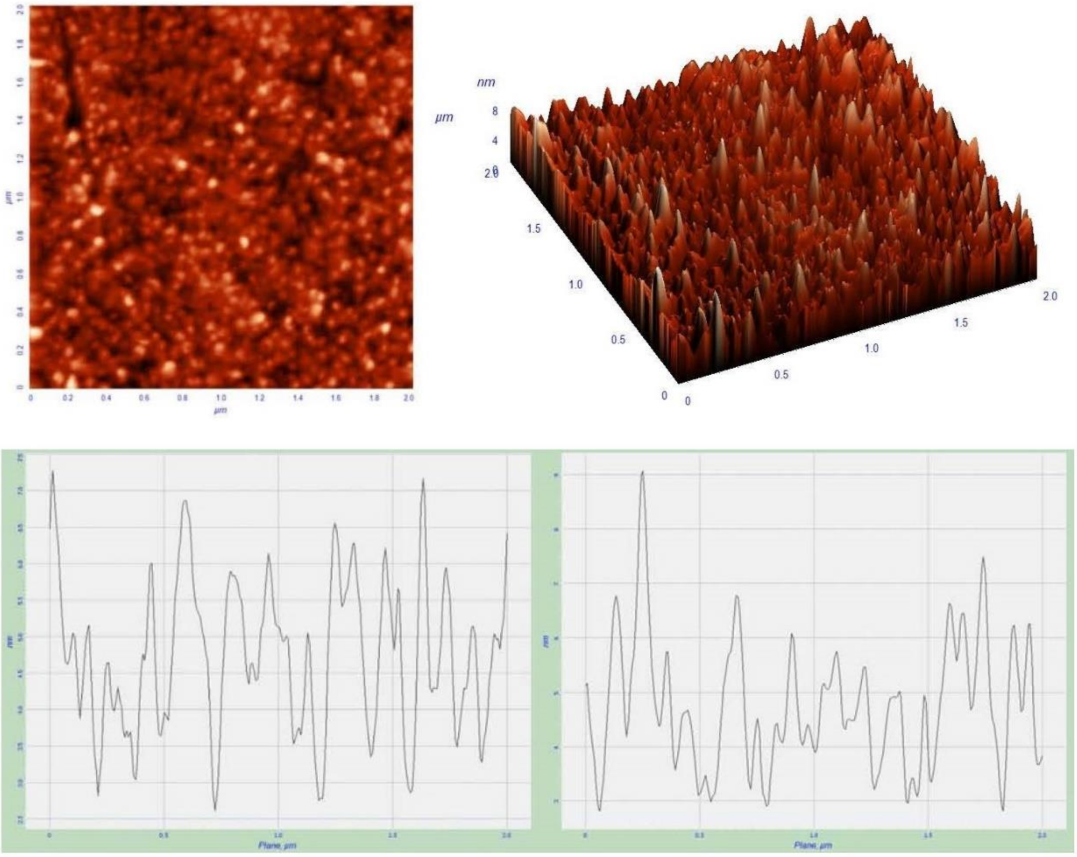

Figure S5. AFM image of the gold sensor with the height profile. The three main parameters characterizing the surface topography, i.e., the average surface height $\bar{h}$, the root mean square (*rms*) and the skewness (*sk*) were calculated as follows [11,12].

$$\bar{h} = \frac{1}{N_i}\sum_{i=1}^{N_i}\left(h_i - h_0\right)$$

$$rms^2 = \frac{1}{N_i}\sum_{i=1}^{N_i}\left(h_i - \bar{h}\right)^2 \tag{S3}$$

$$sk = \frac{1}{rms^3 N_i}\sum_{i=1}^{N_i}\left(h_i - \bar{h}\right)^3$$

where h_i are the surface heights at the consecutive pixels, h_0 is the reference (minimum) height and N_i is the number of pixels corresponding to the AFM scanning area.

These and other relevant parameter such as the maximum surface height, the correlation lengths reflecting the roughness lateral size and the wavelength corresponding the average distance among the roughness peaks are collected in Table S1.

Table S1. Topographical parameters of the gold sensor derived from the AFM measurements.

**Parameter, symbol**	**Value**
Average surface height, $\bar{h}$<br>Maximum surface height, h_{mx}	4.0±0.3 nm<br>5.0±0.5 nm
rms factor, *rms*	1.4±0.1 nm
skewness, *sk*	0.30±0.05
roughness correlation length, λ_w	50±5 nm
roughness wavelength, ξ_w	70±5 nm
maximum roughness wavelength, ξ_{wmx}	200±20 nm

4. Modeling Deposition Kinetics –the Hybrid RSA Approach

Particle deposition kinetics under convective-diffusion transport conditions at solid substrates (for example QCM sensor) can be theoretically described using a hybrid approach exploiting the convective-diffusion equation [13,14]

$$\frac{\partial n}{\partial t} = D\,\nabla^2 n - \frac{D}{kT}\nabla\cdot(\mathbf{F}\,n) - \mathbf{V}\cdot\nabla n \qquad \text{(S4)}$$

where n is the number concentration of particles, t is the time, D is their translational diffusion coefficient, **F** is the external force vector and **V** is the unperturbed (macroscopic) fluid velocity vector.

Eq.(S4) is coupled with the surface layer transport equation where the fluid convection effects are neglected

$$j_a = \frac{dN}{dt} = k_a\, n(\delta_a)\; B(N) - \frac{k_d}{S_g} N \qquad \text{(S5)}$$

where j_a is the net adsorption/desorption flux, N is the particle surface concentration, k_a, k_d are the adsorption and desorption constants, $n(\delta_a)$ is the number concentration of particles at the adsorption boundary layer of the thickness δ_a and $B(N)$ is the generalized blocking function.

Under convective transport, where the particle concentration $n(\delta_a)$ remains in a local equilibrium with the surface coverage, the constitutive expression for the adsorption flux, Eq.(S5) can be expressed as [14]

$$j_a = \frac{K\,B(N) - K_d N}{(K-1)B(N)+1} k_c n_b \qquad \text{(S6)}$$

where $K = k_a / k_c$ is the dimensionless coupling constants, $K_d = k_d / (S_g k_c n_b)$ is the dimensionless desorption constant, k_c is the bulk transfer rate constant, known in analytical form for many types of flows and n_b is the bulk number concentration of particles.

Eqs.(S5,S6) can be expressed in the form of the definite integral

$$\int_{\Gamma_0}^{\Gamma} \frac{\left(k_{\mathrm{a}} - k_c\right) B(Y) + k_c}{k_a c_b B\left(Y\right) - k_d Y} dY = k_{\mathrm{c}} t \qquad \text{(S7)}$$

where $\Gamma = N\, m_1$ is the mass coverage of the particle layer, m_1 is the mass of a single particle Γ_0 is the initial coverage, and $c_b = m_1 n_b$ is the mass concentration of particles in the bulk.

Eq.(S7) represents a general solution for particle deposition kinetics under convection driven transport. It can be evaluated by numerical integration methods if the blocking function is known. However, for bulk transport controlled regime characterized by the condition $k_a >> k_c$ and a lower coverage range, Eq.(S7) simplifies to the linear form

$$\Gamma = k_c c_b t \qquad \text{(S8)}$$

The adsorption and the desorption constants in Eq.(S7) can be calculated from the DLVO energy profiles, if the particle and sensor zeta potential are known, as well as the Hamaker constant [14].

In order to explicitly calculate particle deposition kinetics from Eq.(S7) one should also know the blocking function, which can be conveniently acquired from the random sequential adsorption (RSA) modeling [14-16]. For this purpose it is convenient to express the blocking function in terms of the dimensionless (absolute) particle coverage defined as

$$\Theta = \frac{3}{4\rho_p b}\Gamma \tag{S9}$$

where ρ_p is the particle density and *b* is the shorter semi-axis of the spheroid, or the spheroid particle radions.

Using this definition, one can approximate the blocking function for not too large coverage range, by the second order series expansion [15,16]

$$B(\Theta) = 1 - C_1\Theta + C_2\Theta^2 + 0(\Theta^3) \tag{S10}$$

For spheres one has $C_1 = 4$ and $C_2 = 6\sqrt{3}/\pi = 3.31$.

For prolate spheroids adsorbing side-on, the constants can be calculated from the following expression [14]

$$\begin{aligned} C_1 &= \frac{4(\pi^2 - 4)}{\pi^2} + \frac{8}{\pi^2}\left(As + As^{-1}\right) \\ C_2 &= q_s(As)\, C_1^2 \\ q_s(As) &= 0.126 + 0.181\left(\frac{1 + As^2}{1 + 0.448As + As^2}\right)^4 \end{aligned} \tag{S11}$$

where $As = a/b$ is the longer to shorter axis ratio of the spheroid .

Eq.(S11) yields a few per cent precision for $1 < As < 15$.

The blocking function can also be calculated for a broader coverage range exploiting the scaled particle theory (SPT) [17] enabling to derive the following general expression [14]

$$B(\Theta)=(1-\Theta)e^{-(1+2\gamma_p)\frac{\Theta}{1-\Theta}-\gamma_p\left(\frac{\Theta}{1-\Theta}\right)^2} \tag{S12}$$

where $\gamma_p = \dfrac{P_r^2}{4\pi S_g}$ is the particle shape parameter and P_r is its perimeter.

For spheres γ_p = 1, whereas for prolate spheroids the γ_p parameter can be calculated as [15]

$$\gamma_p = \frac{4As}{\pi^2} E_l^2\left(\frac{As^2-1}{As^2}\right) \tag{S13}$$

where

$$E_l(x) = \int_0^{\frac{\pi}{2}} \left(1 - x\sin^2\xi\right)^{1/2} d\xi \tag{S14}$$

is the complete elliptic integral of the second kind.

One can calculate from these equations that for As = 5, the C_1, C_1 constants are equal to 6.59 and 10.7, whereas γ_p = 2.29.

However, Eq.(S12) derived for equilibrium particle systems, becomes less accurate in the case of irreversible particle deposition where the coverage approaches the jamming limit denoted by Θ_∞. For such a coverage range the blocking function for prolate spheroids adsorbing side-on is given by the asymptotic formula [15]

$$B(\Theta) = C_\infty\left(1 - \frac{\Theta}{\Theta_\infty}\right)^4 \tag{S15}$$

where the dimensionless constant C_∞ varies between 2.8-3.2 for spheroids.

The jamming coverage calculated applying the RSA modeling is equal to 0.547 for spheres and 0.536 or spheroids characterized by As = 5 [14,15].

On the other hand, for spheres the blocking function for the coverages close to the jamming limit, is given by

$$B(\Theta) = 2.31\left(1 - \frac{\Theta}{\Theta_\infty}\right)^3 \tag{S16}$$

In the case of spheres, one can also formulate an analytical expression fitting well the exact numerical data for the entire range of coverage [16]

$$B(\Theta) = \left[1 + 0.812\bar{\Theta} + 0.4258\left(\bar{\Theta}\right)^2 + 0.0716\left(\bar{\Theta}\right)^3\right]\left(1 - \bar{\Theta}\right)^3 \tag{S17}$$

where $\bar{\Theta} = \frac{\Theta}{\Theta_\infty}$.

In Ref. [14] the more general deposition regime was analyzed where the spheroidal particles, except for the side-on orientation, were able to adsorbed under arbitrary orientation, comprising a perpendicular one, if there was enough accessible surface area on the substrate. This regime is likely to appear for long deposition times which results in the significant increase in the jamming coverage described by the formula [14]

$$\Theta_\infty = 0.304 + 0.365As - 0.123As^{-1} \tag{S18}$$

For $As = 5$ one can predict from Eq.(S18) that $\Theta_\infty = 2.1$, which is considerably larger than the value of 0.536 pertinent to the side-on adsorption regime.

Because of the increase in the jamming limit, the blocking function of prolate spheroids for the coverage range above 0.5 calculated in Ref. [14] was considerably larger than that described by Eq.(S15).

It was also shown that the above results obtained pertinent to hard particles can also be extended to the case of particles interacting via the short-range repulsive Yukawa potential. For electrostatic double-layer interactions the characteristic range of this potential h^* is given by [14]

$$h^* = \frac{1}{2\kappa a}\left[\ln\frac{\phi_o}{\phi_{ch}} - \ln\left(1 + \frac{1}{2\kappa a}\ln\frac{\phi_o}{\phi_{ch}}\right)\right] \tag{S19}$$

where ϕ_o is electrostatic energy at contact and ϕ_{ch} is the characteristic interaction energy.

Consequently, one can calculate the jamming coverage for interacting particles (referred to as the maximum coverage) from the relationship

$$\Theta_{mx} = \Theta_\infty \frac{1}{(1+h^*)^2} \tag{S20}$$

Knowing Θ_{mx} one can use Eq.(S17) to calculate the blocking function substituting $\overline{\Theta} = \frac{\Theta}{\Theta_{mx}}$

5. Calculation of DLVO Energy

According to the DLVO theory, the total interaction energy of particles with solid substrates consist of the van der Waals and the electric contributions appearing because of the presence of double layers. In consequences at distances smaller than the particle shorter semi-axis, the net interaction energy ϕ can be calculated as follows

$$\phi = -G_D \frac{A_{123}}{6\,h} + G_D \pi \varepsilon \left(\frac{kT}{e}\right)^2 f\left(\kappa h\right) \tag{S21}$$

where G_D is the geometrical Derjaguin factor depending on the spheroid shape and orientation, A_{123} is the Hamaker constant describing the van der Waals interaction of the particle through the electrolyte with the surface, h is the surface to surface distance and $f\left(\kappa h\right)$ is the function describing the dependence of the electrostatic interactions on the distance.

For the lower zeta potential range of particles pertinent to our experimental system the $f\left(\kappa h\right)$ function can be expressed in the following analytical form [14]

$$f\left(\kappa h\right) = \left[\left(\overline{\zeta}_1^2 + \overline{\zeta}_2^2\right)\ln\left(1-e^{-2\kappa h}\right) + 2\overline{\zeta}_1\overline{\zeta}_2 \ln\frac{1+e^{-\kappa h}}{1-e^{-\kappa h}}\right] \tag{S22}$$

where $\overline{\zeta}_1 = \zeta_1 \frac{e}{kT}; \overline{\zeta}_2 = \zeta_2 \frac{e}{kT}$ are the normalized zeta potentials of the particle and the substrate, respectively.

The Derjaguin factor depends on the orientation angle ϑ of the longer spheroid axis 2*a* relative to the sensor and is given by the formula [14]

$$G_D = a(b/a)^2 / [(b/a)^2 \cos^2\vartheta + \sin^2\vartheta] \tag{S23}$$

Obviously, for spheres, where *a* = *b*, $G_D = b$ for all orientations.

It should also be mentioned that Eq.(S23) is valid for arbitrary particle/surface separations.

For the side-on spheroid orientation where ϑ= 0, $G_D = a$, for the angle of 30°, $G_D = 0.10\,a$, and for the perpendicular orientation $G_D = a^2/b$. Thus, the ratio of energies for the side-on and the perpendicular (end-on) orientations $\phi_{\parallel}$, $\phi_{\perp}$ is equal to

$$\phi_{\parallel} / \phi_{\perp} = a^2 / b^2 \tag{S24}$$

Therefore, in our case, where *a/b* = 5, the entire energy profile for the side-on orientation is 25 times larger than for the end-on orientation.

Taking the zeta potentials values pertinent to our experimental system, i.e., ζ_1= -49 mV for spheroidal particles, ζ_2= 60 mV for PAH covered surface, κ^{-1} = 9.6 nm (at 1 mM NaCl) and the Hamaker constant of 1.7×10^{-20} J [14] one can calculate that the van der Waals interaction energy at the distance $h = h_m$ = 5 nm (for the side-on orientation of spheroids) is equal to -14 *kT* whereas the electrostatic energy is equal to -2800 *kT*. For spherical particles, where ζ_1= -60 mV (at 1 mM NaCl) the net interaction energy calculated from Eq.(S21) is equal to -680 *kT*. Given that in both cases the energy is strongly negative, particle motion perpendicular to surface because of diffusion is prohibited. This can be quantitatively evidenced considering that the increase in the interaction energy due to by the change in the particle distance by a small increment Δh is given by the following formula

$$\Delta\phi_{\parallel} = G_D \pi \varepsilon \left(\frac{kT}{e}\right)^2 f'(h_m)\Delta h \tag{S25}$$

where

$$f'(\kappa h_m) = 2\kappa \frac{e^{-\kappa h_m}}{1-e^{-2\kappa h_m}}\left[\left(\bar{\zeta}_1^2+\bar{\zeta}_2^2\right)e^{-\kappa h_m} - 2\bar{\zeta}_1\bar{\zeta}_2\right] \tag{S26}$$

In Eq.(S25) the van der Waals term was neglected because it was much smaller in comparison with the electrostatic term.

Therefore, the probability Δp of such a perpendicular distance shift from the minimum distance of 5 nm is given by

$$\Delta p = e^{-\Delta\phi_{\parallel}/kT} \tag{S27}$$

One can calculate using Eqs.(S25, S26 and S27) that for Δh = 0.1 nm, where $\Delta\phi_{\parallel}$ = 49 kT the probability is equal to ca 10^{-21}, i.e., extremely small. Even for Δh = 0.01 nm, where $\Delta\phi_{\parallel}$ = 5 kT, the probability is only 0.007. Equally small probabilities are predicted for the spherical particle.

a)

b)

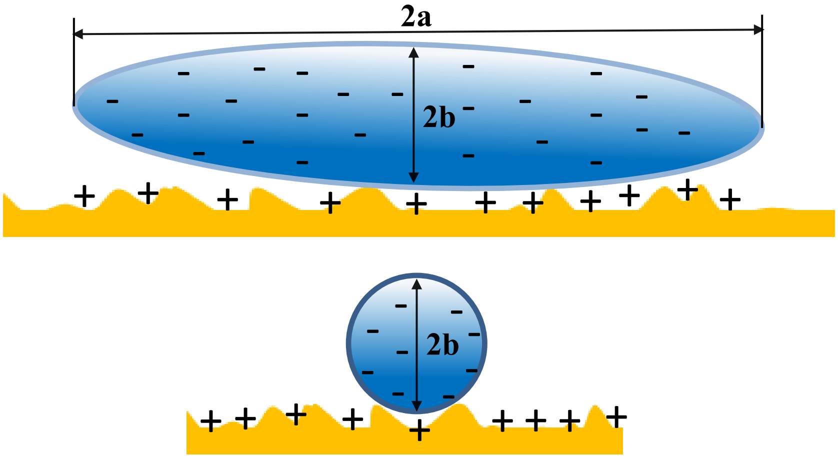

Figure S6. A schematic view of the adsorption of a spheroidal particle (part a) and a spherical particle (part b) at the rough gold sensor.

These estimations unequivocally indicate that the contact of the particles with the sensor can be perfectly stiff in respect to the perpendicular coordinate. However, the particles could move in the parallel direction if the wavelength of the surface roughness was much smaller than the particle size. This condition is met for the spheroidal particles whose length was 1010 nm, whereas the wavelength of the surface roughness was about 200 nm as estimated above (see Figure S4a).

A different situation appears for spherical particles whose diameter practically matches the wavelength, where the deposited particles are irreversibly trapped in local energy minima (see Figure 4b) and their escape probability minimal as estimated above. Therefore, their contact with the sensor could be treated as perfectly stiff.

References

1. Ho, C.C.; Keller, A.; Odell, J.A.; Ottewill, R.H. Preparation of monodisperse ellipsoidal polystyrene particles. *Colloid Polym. Sci*. **1993**, *271 (5)*, 469-479.
2. Komar, P.; Gosecka, M.; Gadzinowski, M.; Gosecki, M.; Makowski, T.; Slomkowski, S.; Basinska, T. Core-shell spheroidal microparticles with polystyrene cores and rich in polyglycidol shells. *Polymer*. **2018**, *146*, 6-11.
3. Fitton, A.O.; Hill, J.; Jane, D.E.; Millar, R. Synthesis of simple oxetanes carrying reactive 2-substituents. *Synthesis*. **1987**, *12*, 1140-1142.
4. Namboodiri, V.V.; Varma, R.S. Solvent-free tetrahydropyranylation (THP) of alcohols and phenols and their regeneration by catalytic aluminum chloride hexahydrate. *Tetrahedron Lett*. **2002**, *43*, 1143-1146.
5. Halacheva, S.; Rangelov, S.; Tsvetanov, C. Poly(glycidol)-based analogues to Pluronic based copolymers. Synthesis and aqueous solution properties. *Macromolecules*. **2006**, *39*, 6845-6852.
6. Gosecka, M.; Slomkowski, S.; Basinska, T.; Chehimi, M.M. Size-controlled 3D colloidal crystals formed in an aqueous suspension of polystyrene/polyglycidol microspheres with covalently bound L-DOPA. *Langmuir*. **2016**, *32*, 12848-12855.
7. Mickiewicz, D.; Gadzinowski, M.; Makowski, T.; Szymański, W.; Slomkowski, S.; Basinska, T. New class of polymer materials - quasi-nematic colloidal particle self-assemblies: the case of assemblies of prolate spheroidal poly(styrene/polyglycidol) particles. *Polymers* **2022**, *14*, 4859.
8. Ohshima, H. Approximate analytic expression for the electrophoretic mobility of moderately charged cylindrical colloidal particles. *Langmuir*. **2015**, *31*, 13633-13638.
9. Morga, M.; Adamczyk, Z. Monolayers of cationic polyelectrolytes on mica-electrokinetic studies. *J. Colloid Interface Sci*. **2013**, *407*, 196-204
10. Morga, M.; Michna, A.; Adamczyk, Z. Formation and stability of polyelectrolyte/polypeptide monolayers determined by electrokinetic measurements. *Colloids Surf. A Physicochem Eng. Asp.* **2017**, *529*, 302-310
11. Pellicione, M.; Karabacak, T.; Gaire, C.; Wang, G.C.; Lu, T.M. Mound formation in surface growth under shadowing. *Phys. Rev. B*. **2006**, *74*, 125420-1-125420-10.
12. Adamczyk, Z.; Sadowska, M.; Nattich-Rak, M. Quantifying nanoparticle layer topography: theoretical modeling and atomic force microscopy investigations. *Langmuir*. **2023**, *39*, 15067-15077.

13. Adamczyk, Z., Kinetics of diffusion-controlled adsorption of colloid particles and proteins. *J. Colloid Interface Sci*. **2000**, *229*, 477-489.
14. Adamczyk, Z., Particles at Interfaces: Interactions, Deposition, Structure. Elsevier; 2017.
15. Ricci, S.M.; Talbot, J.; Tarjus, G.; Viot, P. Random sequential adsorption of anisotropic particles. II. Low coverage kinetics. *J. Chem. Phys*. **1992**, *97*, 5219-5228.
16. Talbot, J.; Tarjus, G.; Van Tassel, P.R.; Viot, P. From car parking to protein adsorption: an overview of sequential adsorption processes. *Colloids Surf. A Physicochem Eng. Asp.* **2000**,*165*, 287-324.
17. Lebowitz, J.L.; Helfand, E.; Praeshaard, E. Scaled particle theory of fluid mixtures. *J. Chem. Phys.* **1965**, *43*, 774-779.
